# Phylogenetic, epidemiological and functional analyses of the *Streptococcus bovis/Streptococcus equinus* complex through an overarching MLST scheme

**DOI:** 10.1186/s12866-016-0735-2

**Published:** 2016-06-21

**Authors:** Christoph Jans, Tomas de Wouters, Bassirou Bonfoh, Christophe Lacroix, Dasel Wambua Mulwa Kaindi, Janine Anderegg, Désirée Böck, Sabrina Vitali, Thomas Schmid, Julia Isenring, Fabienne Kurt, Wambui Kogi-Makau, Leo Meile

**Affiliations:** Laboratory of Food Biotechnology, Institute of Food, Nutrition and Health, ETH Zurich, Schmelzbergstrasse 7, 8092 Zurich, Switzerland; Centre Suisse de Recherches Scientifiques en Côte d’Ivoire (CSRS), KM 17 route de Dabou, Adiopodoumé Yopougon, Abidjan - 01B.P. 1303, Abidjan, Côte d’Ivoire; Department of Food Science, Nutrition and Technology, College of Agriculture and Veterinary Sciences, University of Nairobi, P.O. Box 29053, Nairobi, Kenya

**Keywords:** *Streptococcus infantarius*, *Streptococcus macedonicus*, *Streptococcus gallolyticus*, One health, Adhesion, Inflammation, Colorectal cancer, Foodborne disease, Infective endocarditis, Pathobiont

## Abstract

**Background:**

The *Streptococcus bovis/Streptococcus equinus* complex (SBSEC) comprises seven (sub)species classified as human and animal commensals, emerging opportunistic pathogens and food fermentative organisms. Changing taxonomy, shared habitats, natural competence and evidence for horizontal gene transfer pose difficulties for determining their phylogeny, epidemiology and virulence mechanisms. Thus, novel phylogenetic and functional classifications are required. An SBSEC overarching multi locus sequence type (MLST) scheme targeting 10 housekeeping genes was developed, validated and combined with host-related properties of adhesion to extracellular matrix proteins (ECM), activation of the immune responses via NF-KB and survival in simulated gastric juice (SGJ).

**Results:**

Commensal and pathogenic SBSEC strains (*n* = 74) of human, animal and food origin from Europe, Asia, America and Africa were used in the MLST scheme yielding 66 sequence types and 10 clonal complexes differentiated into distinct habitat-associated and mixed lineages. Adhesion to ECMs collagen I and mucin type II was a common characteristic (23 % of strains) followed by adhesion to fibronectin and fibrinogen (19.7 %). High adhesion abilities were found for East African dairy and human blood isolate branches whereas commensal fecal SBSEC displayed low adhesion. NF-KB activation was observed for a limited number of dairy and blood isolates suggesting the potential of some pathogenic strains for reduced immune activation. Strains from dairy MLST clades displayed the highest relative survival to SGJ independently of dairy adaptation markers *lacS/lacZ*.

**Conclusion:**

Combining phylogenetic and functional analyses via SBSEC MLST enabled the clear delineation of strain clades to unravel the complexity of this bacterial group. High adhesion values shared between certain dairy and blood strains as well as the behavior of NF-KB activation are concerning for specific lineages. They highlighted the health risk among shared lineages and establish the basis to elucidate (zoonotic-) transmission, host specificity, virulence mechanisms and enhanced risk assessment as pathobionts in an overarching One Health approach.

**Electronic supplementary material:**

The online version of this article (doi:10.1186/s12866-016-0735-2) contains supplementary material, which is available to authorized users.

## Background

The *Streptococcus bovis/Streptococcus equinus* complex (SBSEC) is a highly diverse group of bacteria that includes human and animal commensals, opportunistic pathogens and organisms that contribute to traditional food fermentations [1, 2]. The complex currently comprises the species *Streptococcus gallolyticus* subsp. *gallolyticus* (*Sgg*), *Streptococcus gallolyticus* subsp. *pasteurianus* (*Sgp*), *Streptococcus gallolyticus* subsp. *macedonicus* (*Sgm*), *Streptococcus infantarius* subsp. *infantarius* (*Sii*), *Streptococcus lutetiensis* (previously *Streptococcus infantarius* subsp. *coli*), *Streptococcus alactolyticus* and a remaining group of strains originally described as *S. equinus* and *S. bovis* which are allocated to *S. equinus* [1, 2].

SBSEC are commensal colonizers of the human and animal gastrointestinal tract (GI tract). The carriage rate in humans is estimated at 23.8 % in neonates in the UK [3] and around 5 % in adults in France and the UK, which corresponds to the decreasing relative abundance of streptococci in the gut microbiota during aging [3–5]. SBSEC are also highly prevalent among most domesticated and wild animals, including ruminants such as cattle, goats, sheep, deer and camels. Their habitat also extends to bears, piglets, rodents, dogs, sea otters and birds [1].

The SBSEC has been associated with a variety of diseases such as infective endocarditis (IE), bacteremia, biliary tract and prosthetic joint infections as well as meningitis and diarrhea in humans or ruminal acidosis, bloat and laminitis in animals [1]. These diseases are associated with specific (sub)species within the SBSEC such as *Sgg* for IE and bacteremia or *Sgp* and *S. lutetiensis* for infant meningitis, biliary and urinary tract infections [1]. In addition, *S. bovis* has been linked to cancer; specifically *S. bovis* biotype I (=*Sgg*) has been linked to colorectal cancer (CRC) in humans [6] and possibly *S. lutetiensis* to non-colonic cancer [7]. In contrast, *Sgm* and *Sii* play a predominant role in traditional fermented food products of animal and plant origin in Southern Europe, Africa, Asia and North America, indicating the large spectrum of roles and potential public health risks by members of the SBSEC to cause diseases [1, 8, 9].

SBSEC-related factors to cause disease, their associated virulence mechanisms, infection routes, population structure and epidemiology are however not yet elucidated. Pilus proteins encoded in a pil1-operon of *Sgg* are an important virulence factor responsible for adhesion to damaged tissues of heart valves and adenocarcinomas in the colon [10–12]. Furthermore, potential pro-inflammatory proteins [13] were identified in *S. infantarius* NCTC8133 (=CCUG4214), which suggests involvement in CRC development [14]. Shared strain lineages between food products, animals and humans suggest a zoonotic potential and possible infection route via food and fecal-oral transmission [15, 16], which agrees with increased SBSEC-related incidences in rural areas [17]. However, a lack of reliable molecular epidemiological tools and marker genes hinder accurate differentiation of SBSEC subspecies and their individual risk assessments, disease association and the differentiation between commensal and pathogenic subspecies or strain lineages. This difficulty is mainly due to the high degree of gene conservation among members of the SBSEC in combination with evidence of horizontal gene transfer between SBSEC and other streptococci [18, 19], the ability of SBSEC to be naturally competent [19, 20], rapidly changing taxonomy and a broad range of strains from potential food-grade, to commensal and pathogenic organisms fitting the emerging pathobiont concept [21]. The SBSEC therefore requires a holistic approach to elucidate the phylogeny, epidemiology and pathogenicity of its members in relation to their animal, human and food habitats.

Multi locus sequence typing (MLST) has been used to assess global epidemiology and strain lineages based on the combined analysis of short DNA sequences of housekeeping genes [22]. Recently, two such MLST schemes were developed for *Sgg* on different sets of seven housekeeping genes [15, 16]. However, these MLST schemes were not designed to capture the entire SBSEC.

In this study, we aimed to develop an overarching SBSEC MLST scheme based on 10 housekeeping genes. The novel MLST scheme was clustered with phenotypic data of strains, including their survival in simulated gastric conditions, adhesion to extracellular matrix proteins of the human intestinal epithelium and NF-kB activation capacity, all of which are important parameters to evaluate intestinal bacteria and characterize potential pathogens.

## Methods

### Bacterial strains and growth conditions

SBSEC strains (*n* = 74) were used in this study. Of these, 58 originated from the American Type Culture Collection (ATCC, Manassas, VA, USA), the University of Gothenburg (CCUG, Gothenburg, Sweden), Deutsche Sammlung für Mikroorganismen und Zellkulturen (DSMZ, Braunschweig, Germany) and donated by other researchers while 16 strains were analyzed *in silico* using genome sequences available on GenBank [Additional file 1] [23–27]. African strains were selected from our own culture collection (Laboratory of Food Biotechnology, ETH Zurich, Zurich, Switzerland). These strains were isolated from fermented dairy products of cow, camel and goat origin as predominant organisms at 10^8^ colony forming units (CFU) mL^−1^ [Additional file 1] [28–30].

All enterococci and streptococci strains were cultured aerobically overnight in Brain Heart Infusion (BHI, Biolife, Milan, Italy) or M17 broth (Biolife) at 37 °C, except *Streptococcus thermophilus*, which was cultured in M17 broth (Biolife) at 42–43 °C. Lactobacilli were grown anaerobically overnight in MRS Tween 80 broth (Biolife) supplemented with 0.05 % w/w L-cysteine hydrochloride (MRS-C). MRS-C agar was incubated at 37 °C for 2 days. The purity of strains was checked by microscopy, streak plating and rep-PCR fingerprinting as previously described [29]. Stocks were kept in 33 % (v/v) glycerol solution at −80 °C.

All chemicals, proteins and enzymes were obtained from Sigma-Aldrich (Buchs, Switzerland) unless noted otherwise.

### DNA isolation and PCR assays for bacterial identification

DNA was isolated from single colonies on agar media via a short lysis in Triton X-100-based buffer at 95 °C [31]. All primers for PCR amplification and Sanger sequencing were obtained from Microsynth (Balgach, Switzerland). PCR reactions, visualization via gel electrophoresis and analyses of rep-PCR fingerprints were performed as previously described [29]. Sanger sequencing was performed at GATC (Konstanz, Germany) and Microsynth (Switzerland). General 16S rRNA gene amplification was performed using bak11w and bak4 primers [28].

All strains originating from our culture collection were previously identified by rep-PCR fingerprinting, 16S rRNA gene assay and by a PCR assay targeting genes *groES/groEL* for some representative strains [31, 32]. All strains from external sources were identified using a novel *groEL-*sequencing approach, with primers *groEL*-fw and *groEL*-rev for amplification and sequencing that were designed based on ClustalW-aligned *groEL* sequences derived from all available SBSEC genomes [Additional file 2]. These novel primers target conserved binding sites within *groEL* and amplify a 1167-bp product. The g*roEL* PCR assay consisted of 2 min at 95 °C, followed by 35 cycles of 30 s at 95 °C, 30 s at 60 °C, 60 s at 72 °C and final replication for 7 min at 72 °C.

### Presence of *lacS/lacZ* as marker genes for adapted lactose metabolism

*lacS* and *lacZ* with high sequence identity to *S. thermophilus/S. salivarius* were shown to be marker genes for dairy adaptation for African variants of *Sii* [29]. The presence of these two genes in SBSEC strains was assessed and assigned to MLST clusters. Primer pairs lacZ6.2/lacZ-17rev and lacS-8/lacS-18.1rev [Additional file 2] were used as previously described [33], resulting in the amplification of approx. 1 kb of the *lacZ* and 501 bp of the *lacS* gene [33].

### Multi locus sequence typing

#### Selection of target genes/loci

The MLST scheme candidate target genes were first selected and evaluated based on genes used in the MLST-schemes for *Streptococcus pneumoniae, Streptococcus suis, Streptococcus agalactiae, Streptococcus uberis, Streptococcus zooepidemicus* and the salivarius group in addition to several novel candidate loci [Additional file 3] [34–39]. Thirty potential loci for initial *in silico* analysis from SBSEC genomes were identified using CLC Genomic Workbench (version 7.5, Qiagen Aarhus A/S, Denmark). Sequences for each locus were aligned using Bioedit, and compared to establish a phylogenetic tree and a sequence identity matrix using MEGA 5.0 [40]. Ten target loci were chosen on the basis of maximal nucleotide divergence (0.01–16.2 %) within a highly conserved sequence (83.8–100 %) to enable intra- and inter-species differentiation [Additional file 3]. Potential forward and reverse primer binding sites were selected within conserved regions and a distance of approximately 400–500 bp. All primers were designed with a length of 18-22-bp [Additional file 2]. All primers were also used for Sanger sequencing of the amplified DNA fragments. Primers for glucokinase gene amplification were optimized for *S. alactolyticus* using glucokinase sequences of related streptococci to develop degenerate primers. A uniform PCR amplification protocol was used, with an initial denaturation at 95 °C for 3 min, followed by 35 cycles of 30 s each at 95 °C, 59 °C and 72 °C. Final polymerization was performed at 72 °C for 7 min.

#### Establishment of allelic profiles and sequence types for SBSEC isolates

Sequencing reactions for all ten loci were performed on both strands using the same primers as for the initial PCR amplification [Additional file 2]. Sequencing chromatograms were proofread, corrected and assembled using CLC Genomic Workbench. Whole genome sequences of SBSEC isolates obtained from GenBank [Additional file 1] were processed *in silico* using the corresponding SBSEC MLST primers to locate and extract the desired gene sequences. Sequences for each gene were then aligned in MEGA 5.0 using the ClustalW multiple alignment algorithm and trimmed to equal lengths. For each locus, the sequences of all tested isolates were compared and allele numbers were assigned to each unique sequence using CLC Genomic Workbench. Every isolate was hereby defined by a series of ten integers, constituting its allelic profile. Each unique allelic profile was subsequently assigned as the multilocus sequence type (ST) of a strain.

The diversity within each allele was visualized using the SplitsTree 4 software package and aligned sequence files processed by UncorrectedP and Neighbor-Net algorithms [41].

#### Determination of genetic relationships among isolates by computational analysis

The relatedness of isolates was analyzed using the START2 software [42]. START2 was further used to determine the number of polymorphic nucleotide sites, calculate d_N_/d_S_ ratios and construct profile-based dendrograms using the neighbor-joining (NJ) algorithm. Sequence-based trees were constructed in MEGA 5.0 using the NJ algorithm and 1000 bootstrap replications. The classical and standardized index of association (I_A_ and I_A_^S^) were calculated in START2 using only one strain per ST to avoid bias [43]. Additionally, the test of Sawyer (1989) [44] was applied to the synonymous polymorphic sites (PMS) within the alleles at each locus. Related STs were grouped using the START2 and eBURST V3 [45, 46] to form clonal complexes (CC). CCs were defined as groups of two or more independent isolates where each isolate had identical alleles at seven or more loci with at least one other member of the group [47]. This definition was also used to number phylogenetic tree clades and branches.

The allele profiles per locus were used to calculate the Simpson’s index of diversity (SID) [48], where a value close to 1 indicates high diversity and low diversity for values close to 0. Twice the standard deviation of the SID value was used to obtain an approximation for the 95 % confidence intervals as previously described [49].

### Assay for simulated gastric conditions in simulated gastric juice (SGJ)

For simulated gastric juice (SGJ) assays, strains were grown in M17 broth (Biolife), containing lactose as carbon source to mimic the carbon source of milk. *S. alactolyticus* DSM20728^T^, *S. bovis* DSM20480^T^ and *S. equinus* DSM20558^T^ were incubated in BHI broth (Biolife) containing glucose instead of lactose to promote growth. Bacteria cultures were standardized to an OD_600_ of 1.0 using 5 mL of phosphate-buffered saline (PBS, pH 6.0), which is near the optimal pH for growth of SBSEC [50]. The standardized bacteria suspensions were then centrifuged at 6000 × g for 6 min at 20 °C. The pellet was resuspended in 5 mL PBS (pH 6.0) and stored on ice for 30 min.

SGJ was prepared using 1 g of NaCl (Merck), 0.6 g of pepsin from porcine source (Sigma-Aldrich), and 0.2 g peptone from casein (Merck) dissolved in 200 mL distilled H_2_O [51]. pH was adjusted to pH 3.0 or pH 2.5 using 5 M HCl. These pH values were selected within the range of human gastric conditions (pH 1–5) [52] allowing for adequate time-dependent survival assays over the 15 min experiment duration. All SGJ were filter-sterilized (0.2 μm, Sartorius Stedim Biotech GmbH, Goettingen, Germany) and stored at 4 °C. Phosphate buffered saline (PBS) was prepared containing 40 g L^−1^ NaCl (Merck), 1 g L^−1^ KCl (VWR), 7.2 g L^−1^ Na_2_HPO_4_ (Merck), 1.2 g L^−1^ KH_2_PO_4_ (VWR) in distilled H_2_O, and the pH was adjusted to 6.0 using 5 M HCl for low pH-stress conditions. PBS was autoclaved and stored at 4 °C. The assay was performed in sterile 96-well plates (Bioswisstec AG, Schaffhausen, Switzerland) using 270 μL of SGJ or PBS (pH 6.0) per well. The plates were pre-heated to 37 °C and inoculated with 30 μL of the standardized test culture in parallel for all conditions. Plates were incubated in air-tight plastic containers to reduce evaporation. Samples were analyzed after 0, 5, 10 and 15 min of incubation at 37 °C. Enumeration of surviving bacteria was done after serial dilution from 10^−1^ to 10^−6^ and microspotting of 15 μL onto agar medium (M17 or BHI) with a detection limit of 2.8 log_10_ CFU mL^−1^. The assays were performed with two biological replications each comprising three technical replications. *Sii* CJ18 and *Sii* CCUG43820^T^ were used as control strains in each replication. Relative survival rates per strain were calculated where the input is set at 0 log_10_ CFU mL^−1^ and reduction is expressed in negative log-values [53]. For ease of comparison between strains at a given condition, relative survival rates of each strain were normalized using the formula (x_i_-x_mean_)/SD, where x_i_ is the relative survival rate of a strain, x_mean_ the mean relative survival rate of all strains for one condition and SD the standard deviation.

### Assay for bacterial adhesion to extracellular matrix proteins

BHI broth overnight cultures were standardized to an optical density at 600 nm (OD_600_) of 1.0 using PBS (pH 7.5) and centrifuged for 10 min at 3000 × g. The supernatant was discarded and the pellet resuspended in the original volume of PBS (pH 7.5). The bacteria sample was then divided into two equal aliquots and centrifugation was repeated. The supernatant was discarded and one bacteria aliquot per culture was resuspended in PBS (pH 7.5) and acidified PBS (pH 5.5), respectively. Aliquots were kept on ice until use.

Extracellular matrix proteins (ECM) used in this study were collagen type I (rat tail) and type IV (human cell culture), fibrinogen and fibronectin (human cell culture) and the glyco-protein mucin type II (porcine stomach). Bovine serum albumin (BSA) was used as a control protein (Sigma-Aldrich). Stock solutions of ECMs were prepared at a concentration of 10 μg mL^−1^ in PBS (pH 7.5) except BSA and mucin type II at 0.5 mg mL^−1^ in 0.1 M Tris HCl (pH 8.0). MaxiSorp^TM^ 96-well plates (Thermo Fischer Scientific, Reinach, Switzerland) were treated with 100 μL of an ECM stock solution per well [54]. One 96-well plate was filled with 100 μL of PBS (pH 7.5) as a negative adhesion control of the plastic surface. The plates were incubated overnight at 4 °C. Subsequently, the liquid was poured off by plate inversion and dried for 10 min at 65 °C. The plate surface was blocked with 100 μL PBS (pH 7.5) containing 1 % Tween 20, incubated for 1 h at 37 °C and subsequently washed 3 times with 100 μL PBS (pH 7.5) containing 0.05 % Tween 20. Coated plates were stored at 4 °C with 100 μL PBS (pH 7.5) added per well. Bacteria cells (100 μL) resuspended in PBS pH 7.5 or pH 5.5 were then added to the coated 96-well plates. The inoculated plates were gently centrifuged for 10 min at 400 × *g* at room temperature to enable physical contact of the bacteria with the tested proteins, and the plates were then incubated at 37 °C for 1 h. The wells were then washed three times with 100 μL PBS (pH 7.5) containing 0.05 % Tween 20, and adhering bacteria were fixed for 20 min at 65 °C. Staining was performed using crystal violet (100 μL at 1 mg mL^−1^ per well) for 45 min at room temperature, followed by washing three times with 100 μL PBS (pH 7.5). Bacteria-associated crystal violet was then solubilized by adding 100 μL citrate buffer (50 mM, pH 4.0) to each well and incubating for 1 h at 37 °C under constant agitation at 400–500 rpm. The absorbance of crystal violet was measured at 595 nm (BioTeK, PowerWave XS). The normalized cell adhesion of a strain (x_i_) was calculated from the mean of three independent biological repetitions, using the formula (x_i_-x_median_)/SD [55], where x_median_ and SD were obtained from the adhesion measurements of all strains under one condition. Values larger than 1 from the normalized 0 were considered to be adherent.

### Assay for inflammatory potential via NF-kB activation

The activation of nuclear factor k-light-chain-enhancer of activated B cells (NF-kB) was assessed using THP1-Blue™ cells (InvivoGen/Labforce, Muttenz, Switzerland). These cells express all known Toll-like receptors and enable quantification of the NF-KB activation via the SEAP reporter gene. THP1-Blue™ cells were grown in RPMI 1640 supplemented with 2 mM L-glutamine, 50 IU mL^−1^ penicillin, 50 μg mL^−1^ streptomycin and 10 % heat-inactivated fetal calf serum (FCS) in a humidified 10 % CO_2_ atmosphere at 37 °C. All culture media were supplied by Life Technologies (Zug, Switzerland). The assay was performed in 96-well plates containing 90 μL of THP1-Blue™ cells at a concentration of 2 × 10^4^ cells per well. Bacteria cells were prepared by centrifugation for 5 min at 12,000 × *g* of 1-mL aliquots of bacteria cultures standardized to an OD_600_ of 1.0. The supernatant was transferred into a new tube and both supernatant and pellet were stored at −20 °C until use. THP1-Blue™ cells were activated using 10 μL of the supernatant or resuspended pellet in 1 mL PBS. The plates were incubated for 18 to 24 h. NF-kB activation was quantified by adding 10 μL of supernatant of the stimulated THP1-Blue™ cells to 100 μL QUANTI-Blue™ (InvivoGen). After 1 to 8 h of incubation at 37 °C, SEAP activity was quantified at 655 nm as described by the supplier. Normalization was performed as described for the adhesion assay.

### Statistics

Statistical analysis was performed in JMP11.21 (SAS Institute, Cary, NC, USA). Data was first analyzed for normal distribution using the Shapiro-Wilk-W-Test. Non-normal distribution of log-transformed values required the utilization of a Kruskal-Wallis-Test, which was upon rejection of H_0_ further compared in a post-hoc pair-wise Wilcoxon-Test without correction for multiple comparisons [56] in order to reduce type II errors. This was complemented by graphical analysis and detection of outliers by calculating the interquartile range IQ and the defined limitations of lower fence of Q1-1.5*IQ and upper fence Q3 + 1.5*IQ. Strains displaying values outside of the upper or lower fence were considered outliers.

## Results

### Identification of SBSEC (sub)species by *groEL* and 16S rRNA gene sequencing

Partial *groEL* sequencing was performed on all SBSEC strains to confirm species and subspecies status [Additional file 4]. All *groEL* nucleotide sequences were trimmed to an equal length of 772 bp for comparison and neighbor-joining tree construction [Additional file 5]. *Sii* and *S. lutetiensis* had a maximum identity of 94.9–95.4 %, whereas none of the *S. gallolyticus* branch members displayed more than 91 % identity to *Sii*. The *S. gallolyticus* branch was more conserved, yielding 98.8–99.2 % or 97.7–97.9 % between *Sgm* and *Sgg* or *Sgp,* respectively. Intraspecies variations were generally low, yielding 99.3–100.0 % sequence identity among strains of a single subspecies. *S. equinus* JB1 showed highest identity to *Sii* (98.1 %) by *groEL* and 99.9 % to the *S. lutetiensis* by 16S rRNA gene sequence. In addition, CCUG4214 (isogenetic strain of NCTC8133), which was initially classified as *S. bovis* and subsequently reclassified as *Sii* [13]*,* was again reclassified in this study as *S. equinus.* Similarly, *S. bovis* ATCC700338 was reclassified as *Sgp* based on *groEL* and 16S rRNA gene sequences [Additional files 5 and 6]. Strain J2 40–2 isolated from fermented milk from Bangladesh was identified as *Sgm* by *groEL* sequencing, whereas 16S rRNA gene sequence and rep-PCR fingerprinting indicated *S. infantarius/S. equinus* branch [Additional files 5 and 6].

### MLST loci characterization and key performance identifiers

The MLST assay was designed as an overarching scheme to target the entire SBSEC and validate (sub)species assignment (Tables 1 and 2). Aligned and trimmed sequences were between 393 bp and 573 bp in length (Tables 1 and 2). In total, 66 sequence types (ST) were defined for the 74 strains analyzed. The number of alleles ranged from 17 for *tpiA* to 36 for *pyrE* and *mutS2,* while the number of polymorphic sites (PMS) ranged from 36 for *tpiA* to 172 for *mutS* (Tables 1 and 2). The number of alleles and PMS were lower for individual species of the SBSEC (Tables 1 and 2). *tpiA* showed the highest degree of conservation whereas *mutS2* and *pyrE* displayed the highest variability, which was also reflected in the total number of alleles defined.

**Table 1:** Key characteristics of the SBSEC-MLST scheme for the overall SBSEC, *Sii* and *S. lutetiensis*

			SBSEC (*n* = 74)^2^				*Sii* (*n* = 39)				*S. lutetiensis* (*n* = 8)			
Index of association^1^		I_A_	3.132				2.079				2.422			
		I_A_^S^	0.349				0.231				0.269			
Gene	Gene product	A^3^ [bp]	A^3^ [no]	d_N_/d_S_	pms^4^ [No (%)]	SID (95 % CI)^5^	A [no]	d_N_/d_S_	pms [No (%)]	SID	A [no]	d_N_/d_S_	pms [No (%)]	SID
*ddlA*	D-alanine-D-alanine ligase	489	28	0.079	143 (29.2)	0.905 (0.867–0.943)	5	0.171	5 (1)	0.704 (0.648–0.761)	6	0.123	18 (3.7)	0.929 (0.840–1.017)
*gki*	glucokinase	438	27	0.059	119 (27.2)	0.901 (0.858–0.944)	5	0.144	5 (1.1)	0.668 (0.584–0.752)	5	0.072	21 (4.8)	0.893 (0.722–1.064)
*glnA*	glutamine synthetase	396	21	0.039	59 (14.9)	0.906 (0.878–0.934)	7	0.163	8 (2)	0.745 (0.688–0.802)	6	0.064	10 (2.5)	0.929 (0.806–1.052)
*mutS*	DNA mismatch repair ATPase	552	30	0.028	172 (31.2)	0.926 (0.894–0.959)	5	0.066	4 (0.7)	0.752 (0.688–0.815)	5	0.055	39 (7.1)	0.857 (0.704–1.010)
*mutS2*	mismatch repair ATPase	495	36	0.030	159 (32.1)	0.93 (0.890–0.969)	10	0.263	10 (2)	0.762 (0.656–0.869)	5	0.022	44 (8.9)	0.857 (0.704–1.010)
*pheS*	phenylalanyl tRNA synthetase	480	28	0.026	110 (22.9)	0.923 (0.889–0.957)	10	0.009	51 (10.6)	0.776 (0.680–0.872)	4	0.015	18 (3.8)	0.643 (0.301–0.985)
*proS*	prolyl tRNA synthetase	417	28	0.059	128 (30.7)	0.92 (0.887–0.953)	7	0.353	7 (1.7)	0.756 (0.678–0.833)	5	0.106	18 (4.3)	0.786 (0.521–1.051)
*pyrE*	orotate phosphoribosyl transferase	393	36	0.022	97 (24.7)	0.972 (0.961–0.983)	16	0.061	22 (5.6)	0.927 (0.894–0.960)	5	0.013	22 (5.6)	0.857 (0.704–1.010)
*thrS*	threonyl tRNA synthetase	573	31	0.074	168 (29.3)	0.957 (0.941–0.974)	11	0.105	99 (17.3)	0.883 (0.842–0.924)	5	0.061	27 (4.7)	0.857 (0.704–1.010)
*tpiA*	triosephosphate isomerase	408	17	0.119	36 (8.8)	0.894 (0.862–0.927)	4	0.152	3 (0.7)	0.687 (0.637–0.737)	5	0.056	12 (2.9)	0.893 (0.816–0.969)
Mean (95 % CI)						0.924 (0.874–0.973)				0.766 (0.602–0.93)				0.85 (0.682–1.018)

^1^Index of association based on one strain per ST: I_A_: calculated using classical Maynard Smith approach, I_A_^S^: standardized according to Haubold

^2^SBSEC: includes also 3 *S. gallolyticus* subsp*. pasteurianus* and 1 *S. alactolyticus* strains not listed separately

^3^ *A* allele

^4^ *Pms* polymorphic sites

^5^SID (95 % CI): Simpson’s index of diversity with 95 % confidence interval (CI) calculated from SID+/− 2*Standard deviation

^6^no d_N_/d_S_ calculation due to limited allele numbers and differences

Values were calculated for the entire SBSEC and individual (sub)species, including all 10 target genes and Simpson’s index of diversity (SID) as an indication for the differentiation power among strains

**Table 2:** Key characteristics of the SBSEC-MLST scheme for *S. equinus*, *Sgg *and *Sgm*

			*S. equinus* (*n* = 10)				*Sgg* (*n* = 6)				*Sgm* (*n* = 7)			
Index of association^1^		I_A_	2.092				2.093				0.329			
		I_A_^S^	0.232				0.233				0.037			
Gene	Gene product	A^3^ [bp]	A [no]	d_N_/d_S_	pms [No (%)]	SID	A [no]	d_N_/d_S_	pms [No (%)]	SID	A [no]	d_N_/d_S_	pms [No (%)]	SID
*ddlA*	D-alanine-D-alanine ligase	489	8	0.093	52 (10.6)	0.956 (0.894–1.018)	6	0.098	8 (1.6)	1.000 (NaN^7^-NaN)	2	2.070	4 (0.8)	0.286 (-0.092–0.664)
*gki*	glucokinase	438	8	0.061	32 (7.3)	0.956 (0.894–1.018)	3	0.000	6 (1.4)	0.600 (0.215–0.985)	3	−^6^	6 (1.4)	0.714 (0.563–0.865)
*glnA*	glutamine synthetase	396	2	0.000	1 (0.3)	0.200 (-0.104–0.504)	2	0.000	3 (0.8)	0.600 (0.600–0.600)	1	−^6^	0 (0)	0 (0.000–0.000)
*mutS*	DNA mismatch repair ATPase	552	10	0.015	43 (7.8)	1.000 (NaN^7^-NaN)	3	0.037	17 (3.1)	0.600 (0.215–0.985)	4	0.123	6 (1.1)	0.810 (0.630–0.989)
*mutS2*	mismatch repair ATPase	495	10	0.010	28 (5.7)	1.000 (NaN^7^-NaN)	5	0.017	13 (2.6)	0.933 (0.805–1.062)	2	0.000	2 (0.4)	0.571 (0.465–0.678)
*pheS*	phenylalanyl tRNA synthetase	480	8	0.075	14 (2.9)	0.956 (0.894–1.018)	3	0.000	11 (2.3)	0.600 (0.215–0.985)	2	−^6^	1 (0.2)	0.286 (-0.092–0.664)
*proS*	prolyl tRNA synthetase	417	9	0.046	59 (14.1)	0.978 (0.927–1.028)	4	0.000	5 (1.2)	0.800 (0.528–1.072)	1	−^6^	0 (0)	0 .000 (0.000–0.000)
*pyrE*	orotate phosphoribosyl transferase	393	7	0.040	21 (5.3)	0.933 (0.871–0.995)	5	0.000	4 (1)	0.933 (0.805–1.062)	3	0.042	16 (4.1)	0.762 (0.655–0.869)
*thrS*	threonyl tRNA synthetase	573	7	0.000	17 (3)	0.933 (0.871–0.995)	4	0.032	18 (3.1)	0.800 (0.528–1.072)	2	0.096	87 (15.2)	0.286 (-0.092–0.664)
*tpiA*	triosephosphate isomerase	408	3	0.049	7 (1.7)	0.511 (0.200–0.822)	2	0.000	1 (0.2)	0.533 (0.277–0.790)	2	0.000	1 (0.2)	0.476 (0.183–0.769)
Mean (95 % CI)						0.842 (0.307–1.378)				0.74 (0.393–1.087)				0.419 (−0.173–1.011)

^1^Index of association based on one strain per ST: I_A_: calculated using classical Maynard Smith approach, I_A_^S^: standardized according to Haubold

^2^SBSEC: includes also 3 *S. gallolyticus* subsp*. pasteurianus* and 1 *S. alactolyticus* strains not listed separately

^3^ *A* allele

^4^ *Pms* polymorphic sites

^5^SID (95 % CI): Simpson’s index of diversity with 95 % confidence interval (CI) calculated from SID+/− 2*Standard deviation

^6^no d_N_/d_S_ calculation due to limited allele numbers and differences

^7^NaN: maximum diversity, no CI calculcated. Values were calculated for the entire SBSEC and individual (sub)species, including all 10 target genes and Simpson’s index of diversity (SID) as an indication for the differentiation power among strains

SplitsTree analysis of each individual locus provided network-like structures for all ten loci [Additional file 7]. Least-square tree fitting resulted in values >99.899 for all trees. Within each SplitsTree network, separate clusters were distinguishable for the *S. gallolyticus* and *S. infantarius* branch, with *S. alactolyticus* as the most distant species.

The index of association (I_A_) and its standardized version (I_A_^S^) for the overall SBSEC were 3.132 and 0.349, respectively (Tables 1 and 2). I_A_^S^ values calculated for individual groups were 0.037 (*Sgm*), 0.231 (*Sii*), 0.232 (*S. equinus*), 0.233 (*Sgg*), 0.286 (*S. infantarius* branch), and 0.437 (*S. gallolyticus* branch), while *Sgp* comprised too few strains for calculation. Except for *Sgm*, all other groups including SBSEC displayed significant linkage disequilibrium, suggesting recombinatory evolution. Simpson’s Index of Diversity (SID), which indicates the discrimination power of the method, was 0.924 (95 % CI: 0.874–0.973) for the overall SBSEC approach and 0.766 (0.602–0.93) for *Sii* (Tables 1 and 2).

### Definition of clonal complexes

Clonal complexes (CC) are an important classification tool for MLST schemes. CCs were defined for strains that share 7 out of 10 loci using eBURST V3, and these were depicted in a profile-based neighbor-joining tree [Additional file 8]. A total of 10 CCs were determined, which grouped 42 strains and left 32 singletons. CC1 and CC7 were both composed of West African dairy strains, whereas Kenyan and Somali strains were both grouped in CC4 and CC8 [Additional file 8]. CC2 represented the main human pathogen strain cluster that delineated in close relationship with Ivorian dairy cluster CC7. Small CCs comprising two strains each were found for *Sgp* (CC9) and *S. lutetiensis* (CC10). CC3 comprised most of the dairy *Sgm* strains from Africa and Greece, suggesting close relationships among strains of this subspecies. *S. bovis* (CC6) and *S. equinus* (CC5) cannot yet be considered as real CCs, as each CC contained only two versions of the corresponding type strain obtained from different sources. The number of strains did not allow the definition of CCs for *Sgg* or the prediction of a founding strain per CC. The defined CCs 1–10 suggest clear regional relationships among *Sii* dairy strains (CC1, CC4 and CC8) and even across continents (CC3), but also pathogenic relationships concentrated in CC2.

### MLST-based phylogeny of SBSEC and individual (sub)species groups

**Fig. 1:**
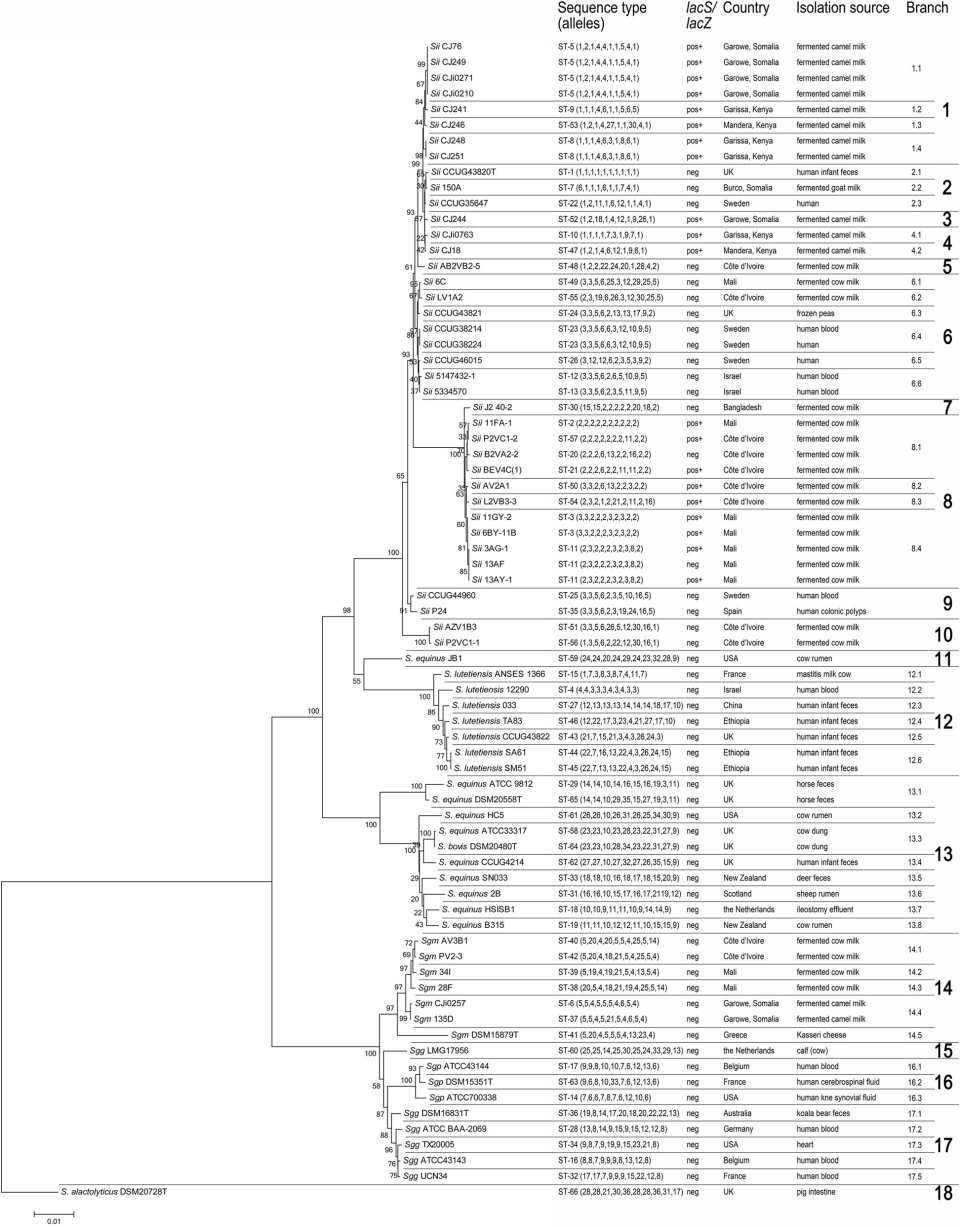
Sequence-based phylogenetic MLST tree of the SBSEC. The MLST tree comprised commensal animal and human strains, human pathogenic strains and food-derived strains of all SBSEC species. The tree was calculated from the concatenated sequences of the 10 MLST loci of each strain using the neighbor joining algorithm and 1000 bootstrap replications. The tree was rooted to *S. alactolyticus* DSM20728^T^. Corresponding STs, alleles and prevalence of dairy adaptation marker genes (*lacS/lacZ*), origin and isolation source are indicated. Branches were defined and numbered according to the clonal complex specifications

The SBSEC MLST scheme provides the first detailed overview of all (sub)species within the SBSEC (Fig. 1). All branches 1–18 were numbered sequentially for ease of comparison using the CC definition of 7 or more identical alleles out of 10.

Primary differentiation established an *S. gallolyticus* clade (branches 14–17) and an *S. equinus/S. lutetiensis/Sii* clade (branches 1–13)*.* Within each clade, species-specific clades were delineated. The *S. equinus* clade (13) harbored both the *S. equinus* and *S. bovis* type strains that originally defined SBSEC (Fig. 1). *S. equinus/S.bovis* strains were distributed between a ruminal/human clade (branches 13.2–13.8) and an equine branch (13.1). *S. equinus* JB1, a ruminant isolate, delineated a separate branch (11) with closer association with the *S. lutetiensis* clade than the major *S. equinus* branch, suggesting reclassification of this strain based on MLST analysis. The *S. lutetiensis* clade reflected a major division between human clade (branches 12.2–12.6) and a potential side branch (12.1) comprising an animal strain (Fig. 1). The human clade was further divided into human blood and fecal isolates.

The *S. gallolyticus* clade showed subdivision into a clade comprising *Sgg* and *Sgp,* and a separate clade of *Sgm* (Fig. 1). Within the *Sgg/Sgp* clade, separate clades were delineated for human pathogenic strains of *Sgg* (branches 17.2–17.3) and *Sgp* (branches 16.1–16.3). Animal commensal strains seemed more heterogeneous, forming two separate branches, one for the *Sgg* type strain DSM16831^T^ (branch 17.1) and one even further related for the calf isolate LMG17958 (branch 15), indicating different lineages of commensal and pathogenic origins. Among the *Sgm* strains, the divisions between Greek (branch 14.5), East African (branch 14.4) and West African (branches 14.1–14.3) strains were fully reflected, suggesting a main African lineage that was further differentiated into East and West African lineages.

The *Sii* clade was analyzed in more detail [Additional file 9]. Several dairy and human clades were delineated, suggesting a general trend of different lineages. Strains of human clinical cases were concentrated in only two specific clades comprising branches 6.4–6.6 and branch 9 regardless of geographic origin. The majority of dairy strains were found in two dairy clades comprising (i) East African dairy isolates (branches 1, 2, 3 and 4) and (ii) West African and Asian dairy isolates (branches 7 and 8), suggesting different lineages within the main dairy lineage of *Sii*. East African dairy strains formed a single clade subdivided into two main clades of dairy isolates only (branch 1) and mixed human and dairy isolates comprising the *Sii* type strain (branches 2–4). The majority of West African and the Asian dairy isolates were grouped in a single dairy clade comprising clades 7 and 8. The delineation of clades 7 and 8 was directly linked to *thrS* alleles 2, 8 and 18 featuring highest sequence identities of 99.1–99.3 % to *S. thermophilus* instead of other *Sii* or SBSEC members as for the other 9 alleles. However, West African dairy strains were more heterogeneous than those of East Africa [Additional file 9], and also featured in addition to clade 8 several shared or related clades with human blood isolates (branches 6 and 10) and East African dairy isolates (branch 5).

### Prevalence of *lacS/lacZ* genes as marker genes for dairy adaptation

The prevalence of *S. thermophilus*-like *lacS/lacZ* genes was assessed as marker genes for dairy adaptations among all SBSEC, including *Sii* strains, using specific PCR assays and previously obtained data [29, 31]. Only *Sii* strains were found to harbor *lacS/lacZ,* with highest prevalence among the major dairy branches 1 (8/8), 3 (1/1), 4 (2/2) and 8 (9/12) (Fig. 1 and [Additional file 9]). In contrast, dairy strains more closely related to human strains, such as *Sii* 150A (branch 2) and the four West African dairy strains found in branches 6 and 10, did not harbor the *lacS/lacZ* genes.

### Resistance to simulated gastric juice (SGJ)

The resistance of SBSEC strains (*n* = 59) to SGJ was tested in SGJ at pH 3.0 or pH 2.5 and PBS at pH 6.0 as control ([Additional files 10, 11 and 12]). All differences in this section were tested for significance at *p* < 0.05 unless mentioned otherwise. Generally, relative cell survival was significantly different for the three conditions pH 2.5 (−log 5.2 ± 1.1 CFU mL^−1^), pH 3.0 (−log 2.7 ± 1.4 CFU mL^−1^) and PBS pH 6.0 (−log 1.2 ± 0.4 CFU mL^−1^) over 15 min. Significantly different survival was also observed among strains at pH 2.5 or pH 3.0 (*p* < 0.05) whereas no significant difference was observed among the strains tested under control conditions of PBS pH6.0 (*p* > 0.05) ([Additional files 10, 11 and 12]). Significantly different survival rates were determined between dairy *Sii* clades 1, 4 and 8 featuring higher survival compared to *Sii* commensal clade 2 and predominantly blood isolate clade 6, *S. lutetiensis* clade 12, *S. gallolyticus* clades 16 and 17 as well as *S. alactolyticus* branch 18 ([Additional files 10 and 12]).

Survival rates were significantly dependent on time-pH combinations. Conditions of pH 2.5 yielded low survival rates, often below detection limit after 15 min ([Additional file 10]). This strong inhibition resulted in a few significant differences between strains and MLST branches with the exception of outliers in clade 8 and branch 12.2. Significantly highest relative survival in comparison to all other strains was determined for the four West African dairy *Sii* strains 6BY-11B, 13AF, 12AY-1 and P2VC1-2, clustered in MLST branches 8.1 and 8.4 as well as the human blood isolate *S. lutetiensis* 12290 (branch 12.2) and all *S. thermophilus* strains ([Additional file 10]). Significantly higher relative survival was therefore determined for West African (branches 8.1 and 8.4) compared to East African *Sii* strains in branches 1 and 2. No significant correlation was observed between *lacS/lacZ* prevalence, habitat and relative survival under the conditions tested (data not shown).

### Adhesion to extracellular matrix proteins

**Fig. 2:**
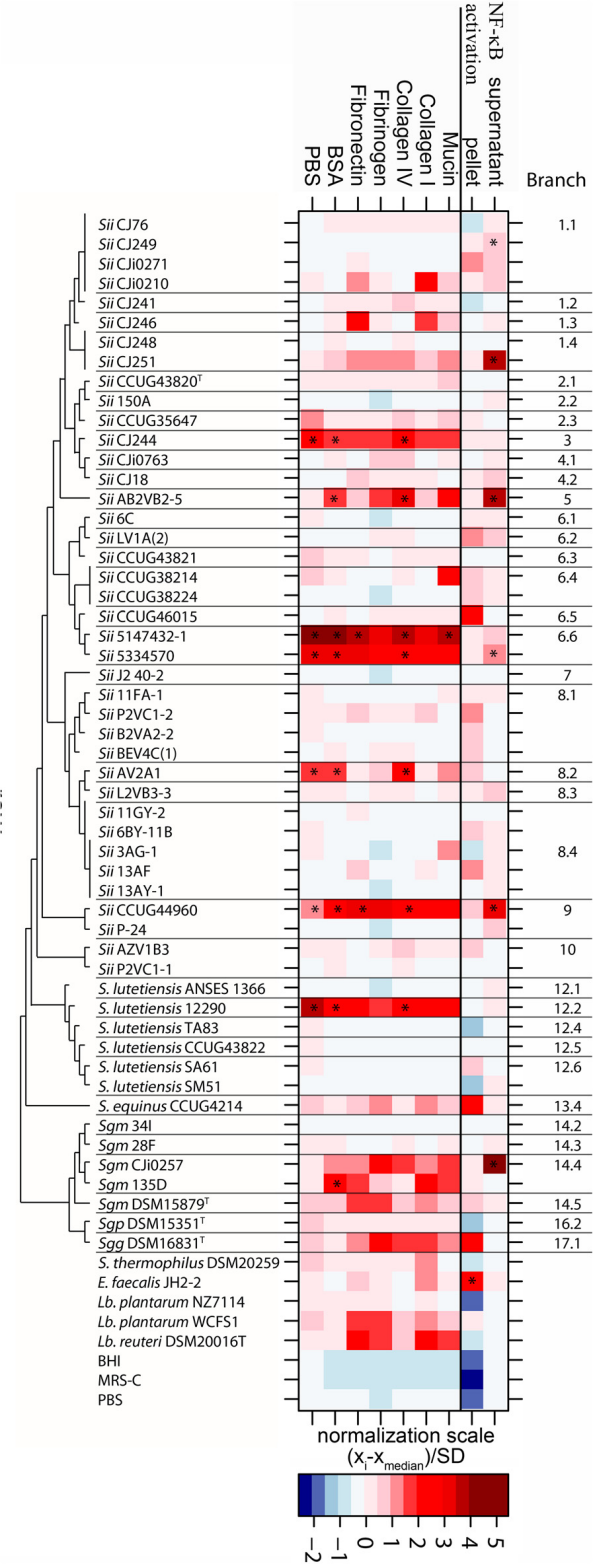
Analysis of the adhesion ability of SBSEC members to extracellular matrix proteins and activation of the NF-kB signaling pathway. Adhesion and NF-kB activation values of individual strains displayed in a heat map. The phylogenetic tree is based on the MLST-tree but not drawn to scale for better visualization. Adhesion measurements were normalized for each strain based on the adhesion median of all strains. Values larger than 1 SD from the normalized 0 adhesion were considered to be adherent. Outliers were marked using an asterix*

Adhesion of SBSEC strains to various ECMs was investigated to estimate their interaction with cell surfaces of the GI tract and blood vessels. Due to heavy tailed distributed values, all adhesion values were normalized and analyzed using a median-based calculation (Fig. 2).

*Lb. plantarum* WCFS1 and *Lb. reuteri* DSM20016^T^ were used as positive adhesion controls showing significant adhesion towards fibronectin, fibrinogen, collagen I and mucin type II that are known factors contributing to their persistence in the human GI tract. As expected, *Lb. plantarum* NZ7114, the sortase knockout mutant of WCSF1 depleted of its adhering surface proteins, showed no adhesion and served as negative adhesion control. Adhesion values obtained at experimental conditions of pH 5.5 showed better reproducibility in contrast to those values obtained at pH 7.5 and were therefore chosen for analysis.

Adhesion characteristics were separated by MLST clustering and isolate origin. Generally, adhesion was most prevalent to the two ECMs collagen I and mucin type II, with 22.6 and 24.5 % of SBSEC isolates showing adhesion (total *n* = 53), respectively, followed by adhesion to fibronectin, fibrinogen and collagen IV by 18.9 % of SBSEC isolates.

Among human *Sii*, adhesion to mucin type II and unspecific adhesion was observed for 44.4 % and adhesion to all remaining ECMs for 33 % of human *Sii* strains (*n* = 9). Among these human strains, comparison of blood isolates (*n* = 4, 5147432-1, 5334570, CCUG38214 and CCUG44960) and potentially commensal isolates (*n* = 5, CCUG35647, CCUG38224, CCUG43820^T^, CCUG46015 and P-24) showed that significant adhesion was exclusively present among *Sii* blood isolates comprised mainly in branches 6.6 and 9. Human fecal isolate *Sii* CCUG43820^T^ featured no significant adhesion abilities to fibronectin, fibrinogen or collagen I and only weak adhesion to collagen IV and mucin type II. Similarly, *S. lutetiensis* blood isolate 12290 (branch 12.2) displayed significant wide range adhesion abilities whereas human fecal isolates of *S. lutetiensis* (branches 12.3–12.6) displayed no significant adhesion abilities, suggesting low prerequisite or other mechanisms contributing to the persistence of these possibly commensal *Sii* and *S. lutetiensis* strains in the human GI tract (Fig. 2).

Similarly, dairy *Sii* (*n* = 29, clades 1–5, 6.1, 6.2, 8 and 10) generally had a low prevalence of adhesion abilities to the ECMs tested and those dairy *Sii* isolates showing adhesion, featured mainly adhesion to collagen IV and mucin type II detected in 13.8 and 17.2 % of strains, respectively, with the exception of CJi0210, CJ246, CJ244 and AB2VB2-5 showing also adhesion to collagen I or mucin type II. Clustered according to MLST and geography, West African dairy *Sii* of MLST branches 8.1, 8.4 and 10 displayed low adhesion abilities to the ECMs tested. Adhesion was more prevalent among East African dairy *Sii* strains with significant adhesion to at least one ECM for strains CJi0210, CJ246, CJ244 and CJ251 as well as West African dairy *Sii* strain AB2VB2-5, which all share the same MLST clade 1–5 (Fig. 2). The significantly adherent West African dairy strain *Sii* AV2A1 was the exception to cluster with the otherwise low-adherent strains of the West African main dairy clade 8 (Fig. 2).

Among the *S. gallolyticus* clade 14–18, high adhesion abilities towards collagen I, IV and fibrinogen was observed. Dairy *Sgm* branches of East Africa (branches 14.2–14.4) and Greece (branch 14.5) showed adhesion towards mucin type II and fibronectin. In contrast, *Sgp* (branch 16.2) did not display any significant adhesion abilities allowing clear separation by MLST clustering from *Sgg* (branch 17.1).

### Determination of immune stimulatory potential by NF-KB activation

Activation of the transcription factor NF-KB was used as marker for the immune stimulation of the SBSEC strains studied (Fig. 2) using the THP1-Blue reporter cell line that expresses all TLRs that detect bacterial motifs in the intestine. NF-KB activation was generally low for the strain panel tested with significantly higher values obtained for cell pellets (mean 0.41, 95 % CI = 0.48–0.55) in contrast to cell-free supernatants (mean 0.05, 95 % CI = 0.12–0.15). Cell pellets, significant inflammation was detected only for individual strains such as *Sii* CCUG46015 (branch 6.5), *S. equinus* CCUG4214 (branch 13.4) and *Sgg* DSM 16831^T^ (branch 17.1). The majority of dairy isolates and also human commensal and blood isolates did not significantly activate NF-KB, particularly in comparison to control strain *E. faecalis* JH2-2. Among cell-free supernatants, highest NF-KB activation was measured for dairy isolates *Sii* AB2VB2-5 (branch 5), *Sii* CJ251 (branch 1.4) and *Sgm* CJi0257 (branch 14.4), as well as human blood isolates *Sii* 5334570 (branch 6.6) and *Sii* CCUG44960 (branch 9), displayed significant NF-KB activation. Again, no significant NF-KB activation was detected among supernatants of other human commensal strains of *S. lutetiensis, Sii* and even pathogenic *S. lutetiensis, Sii*, *Sgg* and *Sgp*.

## Discussion

The SBSEC is a highly diverse group of bacteria colonizing multiple habitats such as food, human and animal GI tracts. Although the 16S rRNA gene sequence provides sufficient power to differentiate the main branches of *S. gallolyticus*, *S. infantarius* and *S. alactolyticus*, differentiation at subspecies level requires sequencing of additional genes such as *groEL* [29, 32]. We showed that *groEL* provided stringent sequence data and discrimination power for SBSEC subspecies identification. Interestingly, *Sii* J2 40-2 seems to be an exception, 16S rRNA gene sequence suggested an *S. infantarius* branch member while *groEL* indicates highest similarity with *Sgm*; however, the identity in the SBSEC MLST scheme was confirmed to be *Sii*. The ongoing horizontal gene transfer (HGT) among streptococci and SBSEC [18, 19] in combination with natural competence [20] may have changed even genes considered as housekeeping genes. Further indications for HGT between dairy *Sii* and *S. thermophilus* were observed among *thrS* alleles of strains comprised in MLST clades 7 and 8 representing Asian dairy strain *Sii* J2 40-2 and the major West African clade. Most strains in clade 8 harbor *lacS* and *lacZ* which serve as marker genes for *S. thermophilus*-like dairy adaptation. In combination with the *thrS* alleles present, this suggests a common ancestor for clades 7 and 8 that underwent multiple HGT with *S. thermophilus*. Ultimately, these observations of HGT and natural competence support the need for a multi gene approach such as MLST for SBSEC (sub)species to ensure accurate identification as well as inter- and intraspecies phylogenetics.

The SBSEC MLST scheme developed in this study provides the first multi-gene-based comparison across the entire SBSEC. In contrast to previous *Sgg* MLST schemes [15, 16], this scheme provides a tool for clustering all currently described SBSEC species and subspecies from human, animal and food sources. The SID of 0.740–0.924 is within the range and confidence interval of that of the *Sgg* MLST (0.84, 95 % CI 0.735–0.931) [15]. Therefore, our novel SBSEC MLST assay provides equally high differentiation power as the *Sgg*-specific assays without the restriction of being limited to a single species but instead being applicable to all (sub)species of the SBSEC. Furthermore, extraction and phylogenetic comparison of MLST alleles from whole genome sequences as performed for several strains in this study ensure the application of this SBSEC MLST scheme also in combination with next generation sequencing of bacterial genomes as data source instead of traditional Sanger sequencing.

The SBSEC MLST scheme provided highly comparable phylogenetic tree delineation for *Sgg* as those obtained via the *Sgg*-specific MLST scheme. d_N_/d_S_ ratios of all loci in the SBSEC MLST scheme were <1 and comparable to those of the two *Sgg*-specific schemes [15, 16]. Number and percentage of PMS of most loci were in the range of Dumke et al. (2014) at 2.23–7.40 % and Shibata et al. (2014) at 6.4–11.1 %. The I_A_ of 2.079–2.271 for individual species was slightly lower than the I_A_ of 2.4 determined by Dumke et al. (2014) whereas Shibata et al. (2014) did not provide a value for I_A_. The I_A_ values for the overall SBSEC, *S. equinus, Sgg, Sii* and *S. lutetiensis* indicated significant linkage disequilibrium between the 10 selected loci as previously observed for the loci set of *Sgg* [15]. Linkage disequilibrium is the effect of non-random association of the selected alleles in an MLST scheme [57], which indicates that recombination was a factor in the evolution of the SBSEC as seen also by network-like structures in SplitsTree analyses [Additional file 7] [37, 41]. *Sgm* was the only subspecies where linkage disequilibrium was not detected. *Sgm* also displayed the lowest genetic diversity despite the analysis of Greek, Somali, Ivorian and Mali strains, suggesting that *Sgm* strains from these different origins are highly related. This could support the theory that *Sgm* is actually a subspecies of dairy origin within the SBSEC [18], enabled through clear delineation from other SBSEC members in this MLST scheme. Genome sequencing of *Sgm* strains will be required to further assess their evolution in relation to the presence of putative *Sgm* dairy adaptation markers such as duplicated *lacG2* described for *Sgm* ACA-DC 198 and decayed or active virulence factors [58].

*Sgg* and *S. lutetiensis* showed a clear differentiation between presumptive human commensal lineages and those of infectious nature isolated from feces, blood or heart samples of humans and animals. In particular, commensal strains such as koala bear isolate *Sgg* DSM16831^T^ and calf isolate *Sgg* LMG17956 were clearly distinct from human pathogenic lineages, suggesting the existence of potentially commensal and pathogenic lineages among many SBSEC species. This requires evaluation in a One Health approach, encompassing animal and human health, food, the zoonotic potential of SBSEC members and their status as pathobionts [21, 59].

The subspecies *Sii* that originated from human and animal-derived food sources had the highest representation in this MLST assay. Clear regional differentiation was observed for the majority of East and West African dairy strains. Differentiation by host and source was observed for most human and dairy strains, suggesting different lineages depending on fecal or blood origin. The MLST phylogeny suggests that human fecal and especially blood isolates cluster by isolation source (feces or blood), and not by geographical origin in contrast to most dairy strains. This possibly reflects different adaptation and risk levels.

Dairy strains clustered in distinct lineages featuring a prominent West African and Asian main branch and an East African branch. The main lineage of East African dairy strains displayed remarkable conservation in contrast to the higher diversity among West African strains and especially those collected in Côte d’Ivoire. The regions of Mandera and Garissa in Kenya share a common cultural background with Somalia. Large Somali populations are represented in both Kenyan regions, with a long tradition in livestock trading, mobile pastoral lifestyle and migration across borders [60, 61], possibly explaining the spreading of similar strain lineages. Certain lineages among the West African dairy *Sii* without the marker genes *lacS/lacZ* clustered separately from the main dairy lineages and closer to human fecal and pathogenic isolates, suggesting the existence of overlapping lineages of *Sii* or contamination of foods by fecal material. This raises concerns about potential health risks of these strain lineages and the origin of these lineages in food.

Exposure of strains to SGJ was used to assess the survival of bacteria in stomach conditions and delivery potential to the colon. Generally, strains of MLST clusters showed similar relative survival being affected by SGJ. However, at pH 3.0 and pH 2.5 several dairy *Sii* clades and mainly four dairy strains showed significantly higher relative survival than human blood *Sii* isolates/clades, respectively, suggesting different adaptation to acidic stress in relation to strain origin and MLST clustering.

MLST-based clustering was further used to assess the ability of individual strains to adhere to ECM and activate the NF-KB pathway in correlation with MLST clades and phylogeny. Adherence is an important first step in colonization of the GI tract or damaged body tissues such as heart valves. MLST clusters provided clear differentiation of highly adhesive *Sii/S. lutetiensis* blood isolates, the *S. gallolyticus* clade, less adhesive East African dairy strains and nearly non-adhesive West African strains, suggesting a generally lower tendency among West African dairy clades and commensal *S. lutetiensis* to adhere to the ECMs tested. The genetic factors contributing to adhesion were so far only related to a specific pilus loci for collagen binding termed *pil1*, *pil2* and *pil3* present in the *Sgg* genomes analyzed [58, 62]. Only *pil3* was found in *Sii*, *Sgm* and *Sgp,* whereas the crucial *pil1* factor of *Sgg* was absent [19, 58]. Comparative genomics and further functional analyses are needed to determine the responsible factors in highly adhering strains of *Sii*, *S. lutetiensis, Sgm* and *Sgg*, and possibly identify new virulence factors to enable an in-depth risk assessment for the different SBSEC species in relation to human and animal diseases.

The NF-KB activation by bacteria attaching to the intestinal mucosa plays a central role in immune signaling of the host, particularly in the intestinal tract. For most strains, NF-KB activation was positively correlated with significant adhesion abilities, suggesting health implications and the need for further functional analyses for such strains. Several blood isolates as well as pathogenic *Sgp* DSM 15351^T^ did not yield significant NF-KB activation, which could enable them to infect the host without inducing a strong immune response [11].

## Conclusions

The overarching MLST scheme for the entire SBSEC provides an epidemiologic and diagnostic tool enabling in-depth inter- and intra(sub)species comparisons of all (sub)species within this emerging human and animal pathogenic complex. The MLST scheme based on 10 housekeeping genes allowed delineating all (sub)species within the SBSEC. It enabled division by host and habitat and furthermore possibly pathogenic lineages from commensal and food lineages. This approach will be useful in enhancing taxonomic classifications of human, animal and food isolates as well as their relationships. Our data suggest that the parallel evolution of East and West African dairy lineages is currently in the process of differentiating from the human lineage including genetic exchange with other streptococci. However, enhanced adhesion abilities of certain lineages similar to those of *Sgg* and the ability to cause inflammation or evade immune response are certainly of concern and require further investigations into pathogenicity mechanisms shared among the SBSEC and those unique to only specific (sub)species. This will greatly assist in designing appropriate treatment strategies for patients infected with this emerging pathogenic group depending on MLST clustering. Furthermore, accurately collecting clinically relevant epidemiologic data in relation to transmission, zoonosis, host specificity and identification of virulence mechanisms will greatly contribute to an enhanced risk assessment of pathogenic, commensal and food lineages or the potential role as pathobionts in an overarching One Health approach.

## Abbreviations

CC, clonal complex; CRC, colorectal cancer; ECM, extracellular matrix protein; GI tract, gastrointestinal tract; HGT, horizontal gene transfer; I_A_, index of association; I_A_^S^, standardized index of association; IE, infective endocarditis; MLST, multi locus sequence typing; PBS, phosphate-buffered saline; PMS, polymorphic sites; SBSEC, *Streptococcus bovis/Streptococcus equinus* complex; *Sgg*, *Streptococcus gallolyticus* subsp. *gallolyticus*; SGJ, Simulated gastric juice; *Sgm*, *Streptococcus gallolyticus* subsp. *macedonicus*; *Sgp*, *Streptococcus gallolyticus* subsp. *pasteurianus*; *Sii*, *Streptococcus infantarius* subsp. *infantarius*; SID, Simpson’s index of diversity; ST, sequence type

## Additional files

Additional file 1: Title of data: Strains used in this study. Description of data: Strains used in this study. (PDF 239 kb) Additional file 2: Title of data: Primers developed and used in this study. Description of data: Primers developed and used in this study for the *Sii*/SBSEC MLST scheme of housekeeping genes and identification based on *groEL, lacS* and *lacZ* genes. (PDF 224 kb) Additional file 3: Title of data: MLST candidate loci used in this study. Description of data: All 30 candidate loci which were used in this study were selected from literature and MLST.net (http://www.mlst.net/). Conserved housekeeping genes were selected on the basis of maximal nucleotide divergence for intraspecies differentiation. All loci which are marked with an asterisk were used for the final SBSEC MLST scheme. (PDF 183 kb) Additional file 4: Title of data: Sequence identity matrix of 772-bp *groEL* fragments of SBSEC species. Description of data: Sequence identity matrix of 772-bp *groEL* fragments of SBSEC species (PDF 92 kb) Additional file 5: Title of data: *groEL*-based phylogenetic tree of SBSEC strains (*n* = 74) of this study. Description of data: Phylogenetic tree of all 74 SBSEC strains investigated this study based on neighbor-joining 3 calculation of a 772-bp sequence fragment of the *groEL* gene. The scale bar below the tree 4 indicates the evolutionary distance using the number of base substitutions per site as units. 5 Species abbreviations: *S. infantarius* subsp*. infantarius (Sii), S. gallolyticus* subsp*. gallolyticus* 6 *(Sgg), S, gallolyticus* subsp*. macedonicus (Sgm), S. gallolyticus* subsp*. pasteurianus (Sgp)*. (TIF 1216 kb) Additional file 6: Title of data: 16S rRNA gene-based phylogenetic tree of selected SBSEC members. Description of data: Phylogenetic tree of selected SBSEC strains (*n* = 24) of all SBSEC (sub)species based on a 930-bp fragment of the 16S rRNA gene. Calculations were performed using the neighbor-joining algorithm. The scale bar below the tree indicates the evolutionary distance using the number of base substitutions per site as units. Species abbreviations: *S. infantarius* subsp*. infantarius (Sii), S. gallolyticus* subsp*. gallolyticus (Sgg), S, gallolyticus* subsp*. macedonicus (Sgm), S. gallolyticus* subsp*. pasteurianus (Sgp)*. (TIF 1425 kb) Additional file 7: Title of data: SplitsTree visualization of the 10 MLST loci. Description of data: The trees were obtained using the defined allele sequences and calculation by neighbor-joining algorithm. Corresponding alleles of selected reference strains are highlighted: *Sii* CCUG43820^T^ = bright green, *Sgg* DSM16831^T^ = red, *Sgm* DSM15879^T^ = ice blue, *S. lutetiensis* CCUG43822 = yellow, *Sgp* DSM15351^T^ = brown, *S. bovis* DSM20480^T^ = pink, *S. equinus* DSM20558^T=^dark blue, *S. alactolyticus* DSM20728^T^ = orange. (TIF 3424 kb) Additional file 8: Title of data: Profile-based MLST tree of all species within the SBSEC. Description of data: The strain pool comprises commensal animal and human strains, human pathogenic strains and food-derived strains. The tree was calculated by neighbor joining algorithm using the allele profiles of 10 housekeeping genes. Corresponding sequence types (ST), alleles and prevalence of dairy adaptation marker genes (*lacS/lacZ*) including origin and isolation source are indicated. Species abbreviations: *S. infantarius* subsp*. infantarius (Sii), S. gallolyticus* subsp*. gallolyticus (Sgg), S, gallolyticus* subsp*. macedonicus (Sgm), S. gallolyticus* subsp*. pasteurianus (Sgp)*. (TIF 2600 kb) Additional file 9: Title of data: Sequence-based phylogenetic MLST tree of *S. infantarius* subsp. *infantarius* (*Sii*). Description of data: The MLST tree comprised commensal animal and human strains, human pathogenic strains and food-derived strains of all SBSEC species. The tree was calculated by neighbor joining algorithm using the concatenated partial sequences of 10 housekeeping genes. Trees were rooted to *S. equinus* JB1. Corresponding sequence types (ST), alleles and prevalence of dairy adaptation marker genes (*lacS/lacZ*) including origin and isolation source are indicated. Branches were defined and numbered according to the clonal complex specifications. (TIF 1167 kb) Additional file 10: Title of data: Relative survival of SBSEC members under simulated gastric conditions at pH 2.5. Description of data: Relative survival of SBSEC strains after 5, 10 and 15 min incubation in SGJ at pH 2.5 performed in two biological replications. Relative survival values in log_10_ CFU mL^−1^ were normalized according to the mean of all measurements of one condition to allow comparison between strains. Positive normalized values indicate relative survival higher than the mean of all strains. Statistical distribution indicators: Median: large black dotted line; Q1 and Q3: small black dotted line; lower and upper outlier fence: large yellow dotted line (only drawn if within the graph range displayed). The phylogenetic tree is based on the MLST-tree but not drawn to scale. (TIF 3894 kb) Additional file 11: Title of data: Relative survival of SBSEC members under simulated gastric conditions in PBS pH 6.0. Description of data: Relative survival of SBSEC strains at 5, 10 and 15 min in PBS pH 6.0 performed in 2 biological replications. Input of 0 log_10_ CFU mL^−1^ was used as basis from which relative survival is expressed. Relative survival values were normalized to allow comparison between strains where positive values indicate relative survival higher than the mean of all strains. Statistical distribution indicators: Median: large black dotted line; Q1 and Q3: small black dotted line; lower and upper outlier fence: large yellow dotted line (only drawn if within the graph range displayed). The phylogenetic tree is based on the MLST-tree but not drawn to scale. (TIF 3405 kb) Additional file 12: Title of data: Relative survival of SBSEC members under simulated gastric conditions at pH 3.0. Description of data: Relative survival of SBSEC strains at 5, 10 and 15 min in in simulated gastric conditions at pH 3.0 performed in 2 biological replications. Input of 0 log_10_ CFU mL^−1^ was used as basis from which relative survival is expressed. Relative survival values were normalized to allow comparison between strains where positive values indicate relative survival higher than the mean of all strains. Statistical distribution indicators: Median: large black dotted line; Q1 and Q3: small black dotted line; lower and upper outlier fence: large yellow dotted line (only drawn if within the graph range displayed). The phylogenetic tree is based on the MLST-tree but not drawn to scale. (TIF 3007 kb)
