# Supplementary material for: Phylogenetic, epidemiological and functional analyses of the Streptococcus bovis/Streptococcus equinus complex through an overarching MLST scheme
Source: BMC Microbiol. 2016 Jun 21;16:117. doi: 10.1186/s12866-016-0735-2 (PMC4915170; doi:10.1186/s12866-016-0735-2)
Supplement: Additional file 1: — Title of data: Strains used in this study. Description of data: Strains used in this study. (PDF 239 kb) [file 12866_2016_735_MOESM1_ESM.pdf]

Additional File 1: Table. Strains used in this study.

Species (original designation)	SBSEC (sub)species according to *groEL* sequence	Strain(s)	Country and place of origin	Isolation source	GenBank accession number	Reference
Enterococcus faecalis	*n/a*	JH2-2	UK	clinical specimen	AXOI01000000	[1]
Lactobacillus rhamnosus	*n/a*	GG (ATCC53103)	USA	feces of a healthy human	AP011548	[2]
Lactobacillus reuteri	*n/a*	DSM20016^T	Germany	intestine of adult		[3]
Lactobacillus plantarum	*n/a*	WCFS1	UK	human saliva, single colony isolate of NCIMB8826,	AL935263	[4]
Lactobacillus plantarum	*n/a*	NZ7114	see WCFS1	sortaseA knockout mutant of WCFS1		[5]
S. alactolyticus	*S. alactolyticus*	DSM20728^T	UK	pig intestine		[6]
S. bovis	*S. equinus*	ATCC33317^T DSM20480^T	UK	cow dung	AUZG01000000	[7, 8]
S. bovis	*Sgp*	ATCC700338	NY, USA	synovial fluid from the knee of a 70 year-old man with septic arthritis	AEEL00000000	[9], HMP[1)]
S. bovis	*S. lutetiensis*	ANSES 1366	Gers, Midi-Pyrenees, France	mastitis milk		[10]
S. bovis	*Sii*: 16S match to *S. infantarius* group; MLST within *Sii* *groEL*= match to *Sgm*	J2 40-2	Bangladesh	fermented cow milk		[11]
S. equinus	*S. equinus*	ATCC9812^T DSM20558^T	UK	horse feces	GL698429	[12]
S. equinus	*S. equinus*	HC5	Ithaca, NY; USA	cow rumen	JPGC01000000	[13]
S. equinus	*S. equinus*	HSISB1	The Netherlands	ileostomy effluent	ASKA01000000	[14]
S. equinus	*S. equinus*	JB1	Davis, CA, USA	cow rumen	AUZH01000000	[15, 7]
S. equinus	*S. equinus*	SN033	New Zealand	deer feces	NZ_ATWZ01000000	2)

Species (original designation)	SBSEC (sub)species according to *groEL* sequence	Strain(s)	Country and place of origin	Isolation source	GenBank accession number	Reference
S. equinus	*S. equinus*	B315	New Zealand	cow rumen	AUJD01000000	2)
S. equinus	*S. equinus*	2B	Scotland	sheep rumen	JNKQ01000000	2)
Sii	*Sii*	CJ251, CJ18, CJ246, CJ248, CJ241, CJi0763,	Kenya	fermented camel milk	CJ18: CP003295	[16, 17]
Sii	*Sii*	CJ244, CJi0210, CJ249, CJi0271, CJ76, 150A	Somalia	fermented camel milk		[16]
Sii	*Sii*	AB2B2-5, AV2A(1), AV3B(1), AZV1B(3), B2VA2-2, BEV4C(1), L2VB3-3, LV1A(2), P2VC1-1, P2VC1-2,	Côte d'Ivoire	fermented cow milk		[17]
Sii	*Sii*	11FA-1, 11GY-2, 13AF, 13AY-1, 3AG-1, 6BY-11B, 6C,	Mali	fermented cow milk		[18]
Sii	*S. equinus*	CCUG4214	UK	human infant feces		[19, 20]
Sii	*Sii*	CCUG35647	Sweden	human with acute lymphatic leukemia 3)		http://www.ccug.se
Sii	*Sii*	CCUG38214	Sweden	human blood, mechanical valve, asthma		http://www.ccug.se
Sii	*Sii*	CCUG38224	Sweden	human 3)		http://www.ccug.se
Sii	*Sii*	CCUG43820^T	UK	human infant feces		[21]
Sii	*Sii*	CCUG43821	UK	frozen peas		[22]
S. lutetiensis		CCUG43822	UK	human infant feces		[22]
Sii	*Sii*	CCUG44960	Sweden	human blood, 78-year old man		http://www.ccug.se
Sii	*Sii*	CCUG46015	Sweden	human 3)		http://www.ccug.se
S. infantarius group	*S. lutetiensis*	SA61, SM51, TA83	Ethiopia	human infant feces		[23]
Sii	*S. lutetiensis*	12290	Israel	human blood		[24]

Species (original designation)	SBSEC (sub)species according to *groEL* sequence	Strain(s)	Country and place of origin	Isolation source	GenBank accession number	Reference
Sii	*Sii*	5147432-1, 5334570	Israel	human blood		[24]
Sii	*Sii*	P-24	Spain	colonic polyps		[25]
Sgg	*Sgg*	ATCC BAA2069	Germany	human blood, infective endocarditis	FR824043	[26]
Sgg	*Sgg*	ATCC43143	Belgium	human blood	AP012053	[27]
Sgg	*Sgg*	DSM16831^{T}	Australia	koala bear feces		[28]
Sgg	*Sgg*	LMG17956	Netherlands	calf	CCBC010000000	[29]
Sgg	*Sgg*	UCN34	France	human blood, infective endocarditis	NC_013798	[30]
Sgg	*Sgg*	TX20005	USA	heart, human	NZ_GL39173	HMP[1)]
Sgm	*Sgm*	DSM15879^{T}	Greece	Greek kasseri cheese		[31]
Sgm	*Sgm*	135D, CJi0257	Somalia	fermented camel milk		[16]
Sgm	*Sgm*	28F, 34I	Mali	fermented cow milk		[18]
Sgm	*Sgm*	PV2(3)	Côte d'Ivoire	fermented cow milk		[17]
Sgp	*Sgp*	ATCC43144	Belgium	human blood	AP012054	[27]
Sgp	*Sgp*	DSM15351^{T}	France	human cerebrospinal fluid		[32]
S. lutetiensis	*S. lutetiensis*	033	China	infant feces	CP003025	[33]
S. thermophilus	n/a	DSM20259	Weihenstephan, Germany	yoghurt		http://www.dsmz.de

n/a: not applicable; 1) HMP: Human Microbiome Project; 2) DOE Joint Genome Institute - The Hungate 1000. A catalogue of reference genomes from the rumen microbiome. 3) body site/material of strain isolation (e.g. feces or blood) unknown.

References Supporting Additional File 1

1. Jacob AE, Hobbs SJ. Conjugal transfer of plasmid-borne multiple antibiotic resistance in *Streptococcus faecalis* var. *zymogenes*. J Bacteriol. 1974;117:360-72.

2. Silva M, Jacobus NV, Deneke C, Gorbach SL. Antimicrobial substance from a human *Lactobacillus* strain. Antimicrob Agents Chemother. 1987;31:1231-3.

3. Kandler O, Stetter KO, Köhl R. *Lactobacillus reuteri* sp. nov., a new species of heterofermentative lactobacilli. Zentralblatt Bakteriol Parasitenkd Infekt Hyg. 1980;C:264-9.

4. Kleerebezem M, Boekhorst J, van Kranenburg R, Molenaar D, Kuipers OP, Leer R et al. Complete genome sequence of *Lactobacillus plantarum* WCFS1. Proc Natl Acad Sci U S A. 2003;100:1990-5.

5. Remus DM, Bongers RS, Meijerink M, Fusetti F, Poolman B, de Vos P et al. Impact of *Lactobacillus plantarum* sortase on target protein sorting, gastrointestinal persistence, and host immune response modulation. J Bacteriol. 2013;195:502-9.

6. Farrow JAE, Kruze J, Phillips BA, Bramley AJ, Collins MD. Taxonomic studies on *Streptococcus bovis* and *Streptococcus equinus*: description of *Streptococcus alactolyticus* sp. nov. and *Streptococcus saccharolyticus* sp. nov. Syst Appl Microbiol. 1984;5:467-82.

7. Benahmed FH, Gopinath GR, Harbottle H, Cotta MA, Luo Y, Henderson C et al. Draft genome sequences of *Streptococcus bovis* strains ATCC 33317 and JB1. Genome Announc. 2014;2.

8. Shattock PMF. The streptococci of group D; serological grouping of *Streptococcus bovis* and observations on serologically refractory group D strains. J Gen Microbiol. 1949;3:80-92.

9. Grant RJ, Shang WY, Whitehead TR. Isolated septic arthritis due to *Streptococcus bovis*. Clin Infect Dis. 1997;24:1021.

10. Chuzeville S, Puymège A, Madec JY, Haenni M, Payot S. Characterization of a new CAMP factor carried by an integrative and conjugative element in *Streptococcus agalactiae* and spreading in streptococci. PLoS One. 2012;7:e48918.

11. Rashid MH, Togo K, Ueda M, Miyamoto T. Identification and characterization of dominant lactic acid bacteria isolated from traditional fermented milk ‘Dahi’ in Bangladesh. World J Microbiol Biotechnol. 2007;23:125-33.

12. Andrewes FW, Horder TJ. A study of the streptococci pathogenic for man. Lancet. 1906;2:708-13.

13. Mantovani HC, Kam DK, Ha JK, Russell JB. The antibacterial activity and sensitivity of *Streptococcus bovis* strains isolated from the rumen of cattle. FEMS Microbiol Ecol. 2001;37:223-9.

14. Van den Bogert B, Boekhorst J, Herrmann R, Smid EJ, Zoetendal EG, Kleerebezem M. Comparative genomics analysis of *Streptococcus* isolates from the human small intestine reveals their adaptation to a highly dynamic ecosystem. PLoS One. 2013;8:e83418.

15. Russell JB, Baldwin RL. Substrate preferences in rumen bacteria: evidence of catabolite regulatory mechanisms. Appl Environ Microbiol. 1978;36:319-29.

16. Jans C, Bugnard J, Njage PMK, Lacroix C, Meile L. Lactic acid bacteria diversity of African raw and fermented camel milk products reveals a highly competitive, potentially health-threatening predominant microflora. LWT-Food Sci Technol. 2012;47:371-9.

17. Jans C, Kaindi DWM, Böck D, Njage PMK, Kouamé-Sina SM, Bonfoh B et al. Prevalence and comparison of *Streptococcus infantarius* subsp. *infantarius* and *Streptococcus gallolyticus* subsp. *macedonicus* in raw and fermented dairy products from East and West Africa. Int J Food Microbiol. 2013;167:186-95.

18. Wullschleger S, Lacroix C, Bonfoh B, Sissoko-Thiam A, Hugenschmidt S, Romanens E et al. Analysis of lactic acid bacteria communities and their seasonal variations in a spontaneously fermented dairy product (Malian fènè) by applying a cultivation/genotype-based binary model. Int Dairy J. 2013;29:28-35.

19. Biarc J, Nguyen IS, Pini A, Gossé F, Richert S, Thiersé D et al. Carcinogenic properties of proteins with pro-inflammatory activity from *Streptococcus infantarius* (formerly *S. bovis*). Carcinogenesis. 2004;25:1477-84.

20. Sharpe ME, Shattock PM. The serological typing of group D streptococci associated with outbreaks of neonatal diarrhoea. J Gen Microbiol. 1952;6:150-65.

21. Sharpe ME. Group D streptococci in the faeces of healthy infants and of infants with neonatal diarrhoea. J Hyg (Lond). 1952;50:209-28.

22. Schlegel L, Grimont F, Collins MD, Régnault B, Grimont PAD, Bouvet A. *Streptococcus infantarius* sp. nov., *Streptococcus infantarius* subsp. *infantarius* subsp. nov. and *Streptococcus infantarius* subsp. *coli* subsp. nov., isolated from humans and food. Int J Syst Evol Microbiol. 2000;50:1425-34.

23. Birri DJ, Brede DA, Tessema GT, Nes IF. Bacteriocin production, antibiotic susceptibility and prevalence of haemolytic and gelatinase activity in faecal lactic acid bacteria isolated from healthy Ethiopian infants. Microb Ecol. 2013;65:504-16.

24. Lazarovitch T, Shango M, Levine M, Brusovansky R, Akins R, Hayakawa K et al. The relationship between the new taxonomy of *Streptococcus bovis* and its clonality to colon cancer, endocarditis, and biliary disease. Infection. 2013;41:329-37.

25. Romero B, Morosini M-I, Loza E, Rodríguez-Baños M, Navas E, Cantón R et al. Reidentification of *Streptococcus bovis* isolates causing bacteremia according to the new taxonomy criteria: still an issue? J Clin Microbiol. 2011;49:3228-33.

26. Hinse D, Vollmer T, Rückert C, Blom J, Kalinowski J, Knabbe C et al. Complete genome and comparative analysis of *Streptococcus gallolyticus* subsp *gallolyticus*, an emerging pathogen of infective endocarditis. BMC Genomics. 2011;12:400.

27. Lin IH, Liu T-T, Teng Y-T, Wu H-L, Liu Y-M, Wu K-M et al. Sequencing and comparative genome analysis of two pathogenic *Streptococcus gallolyticus* subspecies: genome plasticity, adaptation and virulence. PLoS One. 2011;6:e20519.

28. Osawa R, Fujisawa T, Sly LI. *Streptococcus gallolyticus* sp. nov.; gallate degrading organisms formerly assigned to *Streptococcus bovis*. Syst Appl Microbiol. 1995;18:74-8.

29. Romero-Hernández B, Tedim AP, Sánchez-Herrero JF, Librado P, Rozas J, Muñoz G et al. *Streptococcus gallolyticus* subsp. *gallolyticus* from human and animal origins: genetic diversity, antimicrobial susceptibility, and characterization of a vancomycin-resistant calf isolate carrying a *vanA*-Tn*1546*-like element. Antimicrob Agents Chemother. 2015;59:2006-15.

30. Rusniok C, Couvé E, Da Cunha V, El Gana R, Zidane N, Bouchier C et al. Genome sequence of *Streptococcus gallolyticus*: insights into its adaptation to the bovine rumen and its ability to cause endocarditis. J Bacteriol. 2010;192:2266-76.

31. Tsakalidou E, Zoidou E, Pot B, Wassill L, Ludwig W, Devriese LA et al. Identification of streptococci from Greek Kasseri cheese and description of *Streptococcus macedonicus* sp. nov. Int J Syst Bacteriol. 1998;48:519-27.

32. Poyart C, Quesne G, Trieu-Cuot P. Taxonomic dissection of the *Streptococcus bovis* group by analysis of manganese-dependent superoxide dismutase gene (*sodA*) sequences: reclassification of '*Streptococcus infantarius* subsp. *coli*' as *Streptococcus lutetiensis* sp. nov. and of *Streptococcus bovis* biotype II.2 as *Streptococcus pasteurianus* sp. nov. Int J Syst Evol Microbiol. 2002;52:1247-55.

33. Jin D, Chen C, Li L, Lu S, Li Z, Zhou Z et al. Dynamics of fecal microbial communities in children with diarrhea of unknown etiology and genomic analysis of associated *Streptococcus lutetiensis*. BMC Microbiol. 2013;13:141.
