# Supplementary material for: Phylogenetic, epidemiological and functional analyses of the Streptococcus bovis/Streptococcus equinus complex through an overarching MLST scheme
Source: BMC Microbiol. 2016 Jun 21;16:117. doi: 10.1186/s12866-016-0735-2 (PMC4915170; doi:10.1186/s12866-016-0735-2)
Supplement: Additional file 2: — Title of data: Primers developed and used in this study. Description of data: Primers developed and used in this study for the Sii/SBSEC MLST scheme of housekeeping genes and identification based on groEL, lacS and lacZ genes. (PDF 224 kb) [file 12866_2016_735_MOESM2_ESM.pdf]

Additional File 2: Table. Primers developed and used in this study. Primers were designed for the *Sii*/SBSEC MLST scheme of housekeeping genes and identification based on *groEL, lacS* and *lacZ* genes.

Primer name	sequence (5'-3')[1)]	target gene	amplicon size [bp]	gene used as target in MLST of other streps
ddl-fw	CTT ACG TGA CTT ATG TTG AAG G	D-alanine-D-alanine ligase	522	*S. uberis*
ddl-rev	CCA AAG AAG TGG ATA CAT TGA C			
gki-fw2	TGA ACT GGG CAC ACA CTG	glucokinase	521	*S. pneumoniae*
gki-rev	CTG GGT TYA AGA TGT TTG CG			
gki-fw6.2-ala[2)]	GGG MTC WCC TGG AGC TGT			
gki-rev1.2-ala[2)]	PGC AGC WGA HAC DCC ACC			
glnA-fw	GCT GTT GCA GGT TTG ATC TG	glutamine synthetase	478	group B streptococci
glnA-rev	TAC AGT GCA TAC CTG AAC CG			
groEL-fw	GGT TCA CCA CTT ATC ACW AAT G	*groEL* heat shock protein *cpn*60/*hsp*60	1167	only used for (sub)species identification
groEL-rev	ATC ACC GTC AAG TTC AAG TTC			
lacZ-6.2	TTC CTC AAG AAT CAA ATG CTG	β-galactosidase *lacZ*	1027	only used for *lacZ* amplification and sequencing
lacZ-17rev	CCA CAA GAC CAA ATG ATA ACA C			
lacS-8	GCG TGA CGT GCT TCA GTC	lactose symporter/antiporter *lacS*	501	only used for *lacS* amplification and sequencing
lacS-18.1rev	GAT TGA ATA CAG TTG TTG GTT TG			
mutS-fw	GTG CCA GAT TAY TTC TTC CG	DNA mismatch repair ATPase	581	*S. suis*
mutS-rev	GCT TCC ATC ATT TCC ACC AT			
mutS2-fw	CCA AAT ACT GGT GGT AAG AC	mismatch repair ATPase	529	*Sii*
mutS2-rev	TCA AAC GCT CTG CTT CAT TG			
pheS-fw	GCT AAA TTG GCT GAA ATG CG	phenylalanyl tRNA synthetase	557	group B streptococci
pheS-rev	ACA CGY CCT GGT GAR ATC AT			
proS-fw	CTG CTA ACC TTG AAA TGG CA	prolyl tRNA synthetase	455	*Sii*
proS-rev2	AAC GTA TTC CGC TTC AAA ATC			
pyrE-fw	AGC ATC ACA AAT TGC ATC AG	orotate phosphoribosyl transferase	426	*S. salivarius*
pyrE-rev	ACA TCA GCA CCT TCA CGT TC			
thrtRNA-syn-fw	CCA CCG TAA ACT TGG TAA AG	threonyl tRNA synthetase	606	*S. salivarius*
thrtRNA-syn-rev	TGW GCA TTT TCC CAC ATT TC			
tpi fw3	ATC GCT GGT AAC TGG AAA ATG	triosephosphate isomerase	410	*S. zooepidemicus*
tpi-rev	CGA TAG CCC AGA TTG GTT CG			

[1)] mixed base sites according to IUB code [2)] modified primers to amplify the same gene region in

S. alactolyticus.
