# Supplementary material for: Phylogenetic, epidemiological and functional analyses of the Streptococcus bovis/Streptococcus equinus complex through an overarching MLST scheme
Source: BMC Microbiol. 2016 Jun 21;16:117. doi: 10.1186/s12866-016-0735-2 (PMC4915170; doi:10.1186/s12866-016-0735-2)
Supplement: Additional file 3: — Title of data: MLST candidate loci used in this study. Description of data: All 30 candidate loci which were used in this study were selected from literature and MLST.net (http://www.mlst.net/). Conserved housekeeping genes were selected on the basis of maximal nucleotide divergence for intraspecies differentiation. All loci which are marked with an asterisk were used for the final SBSEC MLST scheme. (PDF 183 kb) [file 12866_2016_735_MOESM3_ESM.pdf]

Additional File 3: Table. MLST candidate loci used in this study. All 30 candidate loci which were used in this study were selected from literature and MLST.net (http://www.mlst.net/). Conserved housekeeping genes were selected on the basis of maximal nucleotide divergence for intraspecies differentiation. All loci which are marked with an asterisk were used for the final SBSEC MLST scheme.

**candidate loci**	**MLST and loci originally selected for organism**	**references**
3-phosphoshikimate-3-carboxyvinyltransferase	*Sii*	this study
glucokinase*	*S. pneumoniae*	[1]
α-keto-β-hydroxylacid reductoisomerase	salivarius group	[2]
amino acid ABC transporter	group B streptococci	[3]
aspartate kinase	*S. suis*	[4]
D-alanine-D-alanine ligase*	*S. uberis*	[5]
DNA polymerase III	salivarius group	[6]
endopeptidase O	salivarius group	[6]
glutamate racemase	*S. pyogenes*	[7]
glutamine synthetase*	group B streptococci	[3]
L-serine dehydratase, β subunit	group B streptococci	[3]
L-serine dehydratase, α subunit	group B streptococci	[3]
groEL, hsp60 chaperone	*S. uberis*	[8]
methionine aminopeptidase	*Sii*	this study
DNA mismatch repair ATPase*	*S. suis*	[4]
mismatch repair ATPase*	*Sii*	this study
orotate phosphoribosyltransferase*	salivarius group	[2]
peroxidase resistance gene	*S. pneumoniae*	[1]
phenylalanyl tRNA synthetase*	group B streptococci	[3]
prolyl tRNA synthetase*	*Sii*	this study
recombination protein A	*Sii*	this study
ribonucleoside-diphosphate reductase, major subunit	*S. zooepidemicus*	[9]
ribonucleoside-diphosphate reductase, minor subunit	*S. zooepidemicus*	[9]
shikimate/quinate dehydrogenase	*S. pneumoniae*	[1]
signal peptidase I	*S. pneumoniae*	[1]
threonyl tRNA synthetase*	salivarius group	[6]
thymidine kinase	*S. uberis*	[5]
transketolase	*S. vestibularis*	[6]
triosephosphate isomerase*	*S. zooepidemicus*	[9]
xanthine phosphoribosyltransferase	*S. pneumoniae*	[1]

References supporting Additional File 3

1. Enright MC, Spratt BG. A multilocus sequence typing scheme for *Streptococcus pneumoniae*: identification of clones associated with serious invasive disease. Microbiology. 1998;144:3049-60.

2. Delorme C. Safety assessment of dairy microorganisms: *Streptococcus thermophilus*. Int J Food Microbiol. 2008;126:274-7.

3. Jones N, Bohnsack JF, Takahashi S, Oliver KA, Chan MS, Kunst F et al. Multilocus sequence typing system for group B *Streptococcus*. J Clin Microbiol. 2003;41:2530-6.

4. King SJ, Leigh JA, Heath PJ, Luque I, Tarradas C, Dowson CG et al. Development of a multilocus sequence typing scheme for the pig pathogen *Streptococcus suis*: identification of virulent clones and potential capsular serotype exchange. J Clin Microbiol. 2002;40:3671-80.

5. Coffey TJ, Pullinger GD, Urwin R, Jolley KA, Wilson SM, Maiden MC et al. First insights into the evolution of *Streptococcus uberis*: a multilocus sequence typing scheme that enables investigation of its population biology. Appl Environ Microbiol. 2006;72:1420-8.

6. Delorme C, Poyart C, Ehrlich SD, Renault P. Extent of horizontal gene transfer in evolution of streptococci of the salivarius group. J Bacteriol. 2007;189:1330-41.

7. Enright MC, Spratt BG, Kalia A, Cross JH, Bessen DE. Multilocus sequence typing of *Streptococcus pyogenes* and the relationships between *emm* type and clone. Infect Immun. 2001;69:2416-27.

8. Zadoks RN, Schukken YH, Wiedmann M. Multilocus sequence typing of *Streptococcus uberis* provides sensitive and epidemiologically relevant subtype information and reveals positive selection in the virulence gene *pauA*. J Clin Microbiol. 2005;43:2407-17.

9. Webb K, Jolley KA, Mitchell Z, Robinson C, Newton JR, Maiden MCJ et al. Development of an unambiguous and discriminatory multilocus sequence typing scheme for the *Streptococcus zooepidemicus* group. Microbiol-SGM. 2008;154:3016-24.
