# Supplementary material for: Phylogenetic, epidemiological and functional analyses of the Streptococcus bovis/Streptococcus equinus complex through an overarching MLST scheme
Source: BMC Microbiol. 2016 Jun 21;16:117. doi: 10.1186/s12866-016-0735-2 (PMC4915170; doi:10.1186/s12866-016-0735-2)
Supplement: Additional file 4: — Title of data: Sequence identity matrix of 772-bp groEL fragments of SBSEC species. Description of data: Sequence identity matrix of 772-bp groEL fragments of SBSEC species (PDF 92 kb) [file 12866_2016_735_MOESM4_ESM.pdf]

Additional File 4: Table. Sequence identity matrix of 772-bp *groEL* fragments of SBSEC species.

	Sequence identity range [%]					
	Sii	*S. lutetiensis*	*S. equinus*	*Sgg*	*Sgp*	*Sgm*
Sii	99.3-100.0	94.9-95.4	91.9-93.3 (98.1)[1]	90.1-90.6	90.4-90.7	90.1-90.5
S. lutetiensis		99.4-100.0	92.2-93.6 (95.5)[1]	90.7-91.0	91.1-91.4	90.6-91.0
S. equinus			(92.8)[1] 97.5-100.0	(90.6)[1] 91.6-93.5	(91.3)[1] 92.2-93.3	(90.6)[1] 91.5-93.1
Sgg				99.2-100.0	98.1-98.7	98.8-99.2
Sgp					100.0	97.7-97.9
Sgm						99.8-100.0

[1] Sequence identity value for *S. equinus* JB1 is given in () due to its high divergence within its species
