# Supplementary figures and images for: Phylogenetic, epidemiological and functional analyses of the Streptococcus bovis/Streptococcus equinus complex through an overarching MLST scheme
Source: BMC Microbiol. 2016 Jun 21;16:117. doi: 10.1186/s12866-016-0735-2 (PMC4915170; doi:10.1186/s12866-016-0735-2)

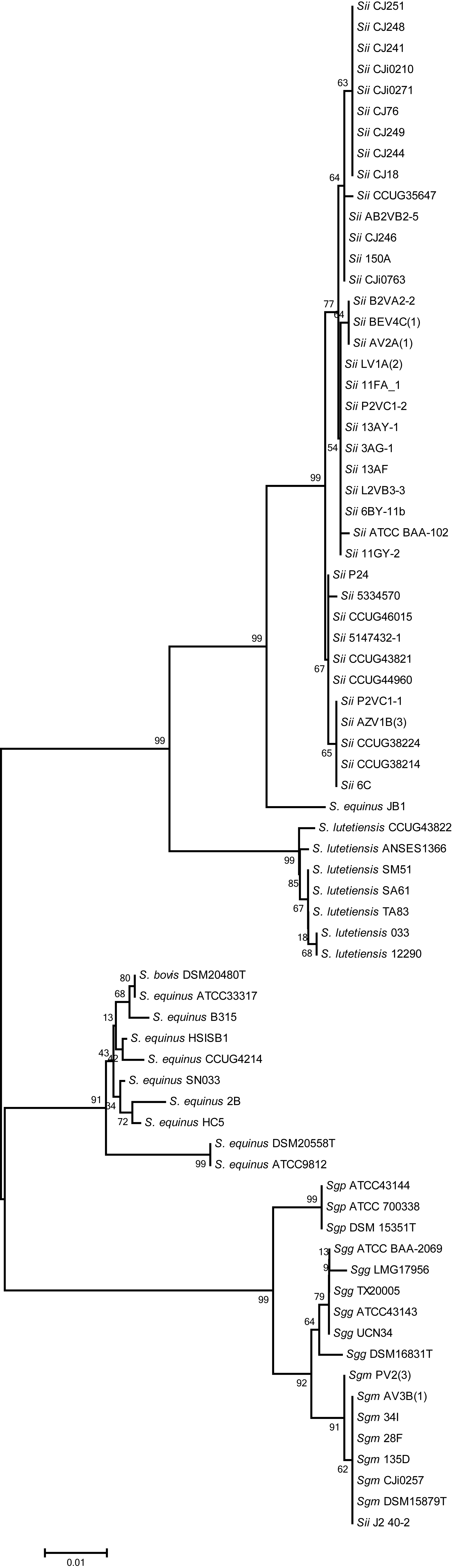

Supplement: Additional file 5: — Title of data: groEL-based phylogenetic tree of SBSEC strains (n = 74) of this study. Description of data: Phylogenetic tree of all 74 SBSEC strains investigated this study based on neighbor-joining 3 calculation of a 772-bp sequence fragment of the groEL gene. The scale bar below the tree 4 indicates the evolutionary distance using the number of base substitutions per site as units. 5 Species abbreviations: S. infantarius subsp. infantarius (Sii), S. gallolyticus subsp. gallolyticus 6 (Sgg), S, gallolyticus subsp. macedonicus (Sgm), S. gallolyticus subsp. pasteurianus (Sgp). (TIF 1216 kb) [file 12866_2016_735_MOESM5_ESM.tif]

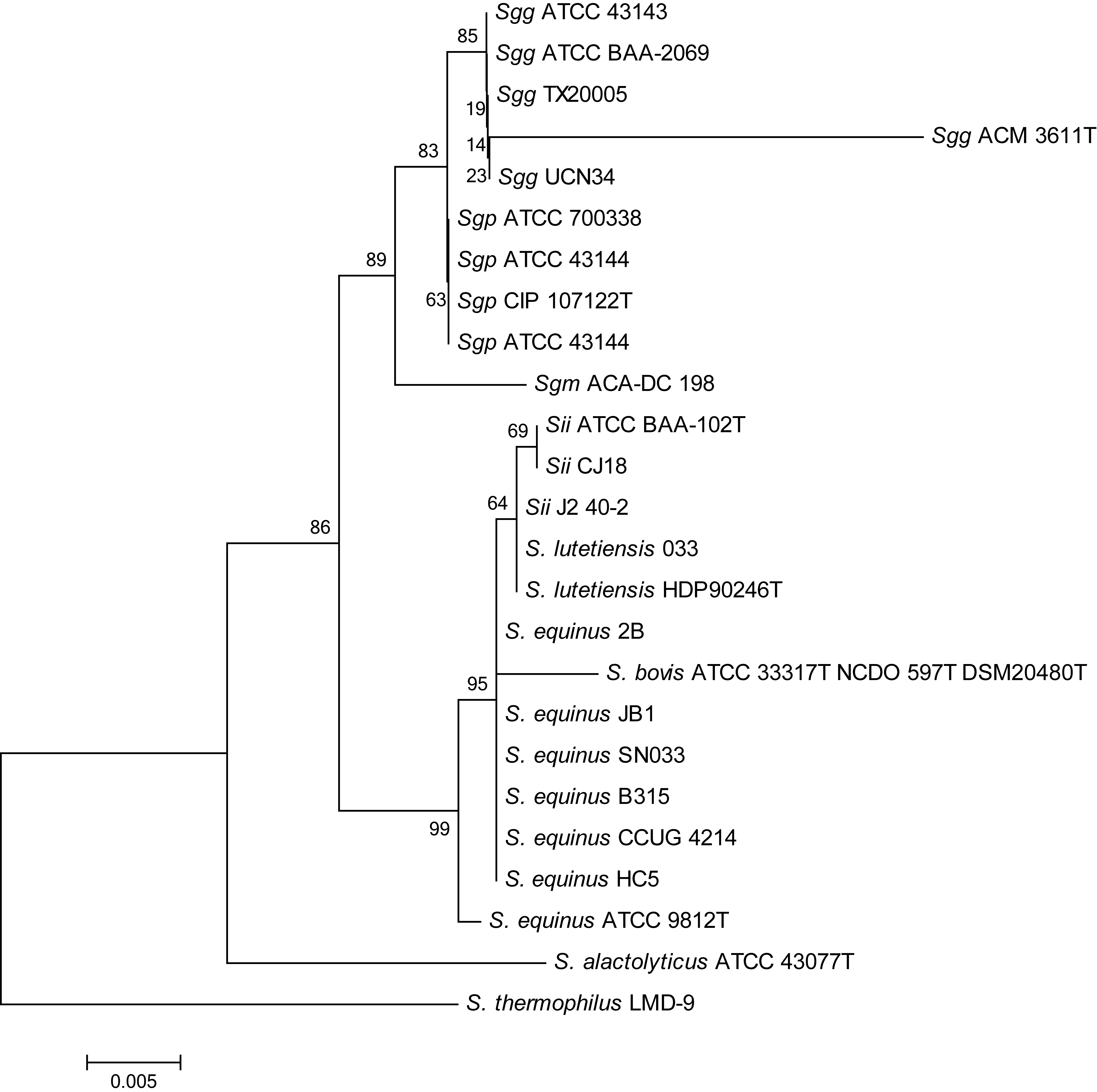

Supplement: Additional file 6: — Title of data: 16S rRNA gene-based phylogenetic tree of selected SBSEC members. Description of data: Phylogenetic tree of selected SBSEC strains (n = 24) of all SBSEC (sub)species based on a 930-bp fragment of the 16S rRNA gene. Calculations were performed using the neighbor-joining algorithm. The scale bar below the tree indicates the evolutionary distance using the number of base substitutions per site as units. Species abbreviations: S. infantarius subsp. infantarius (Sii), S. gallolyticus subsp. gallolyticus (Sgg), S, gallolyticus subsp. macedonicus (Sgm), S. gallolyticus subsp. pasteurianus (Sgp). (TIF 1425 kb) [file 12866_2016_735_MOESM6_ESM.tif]

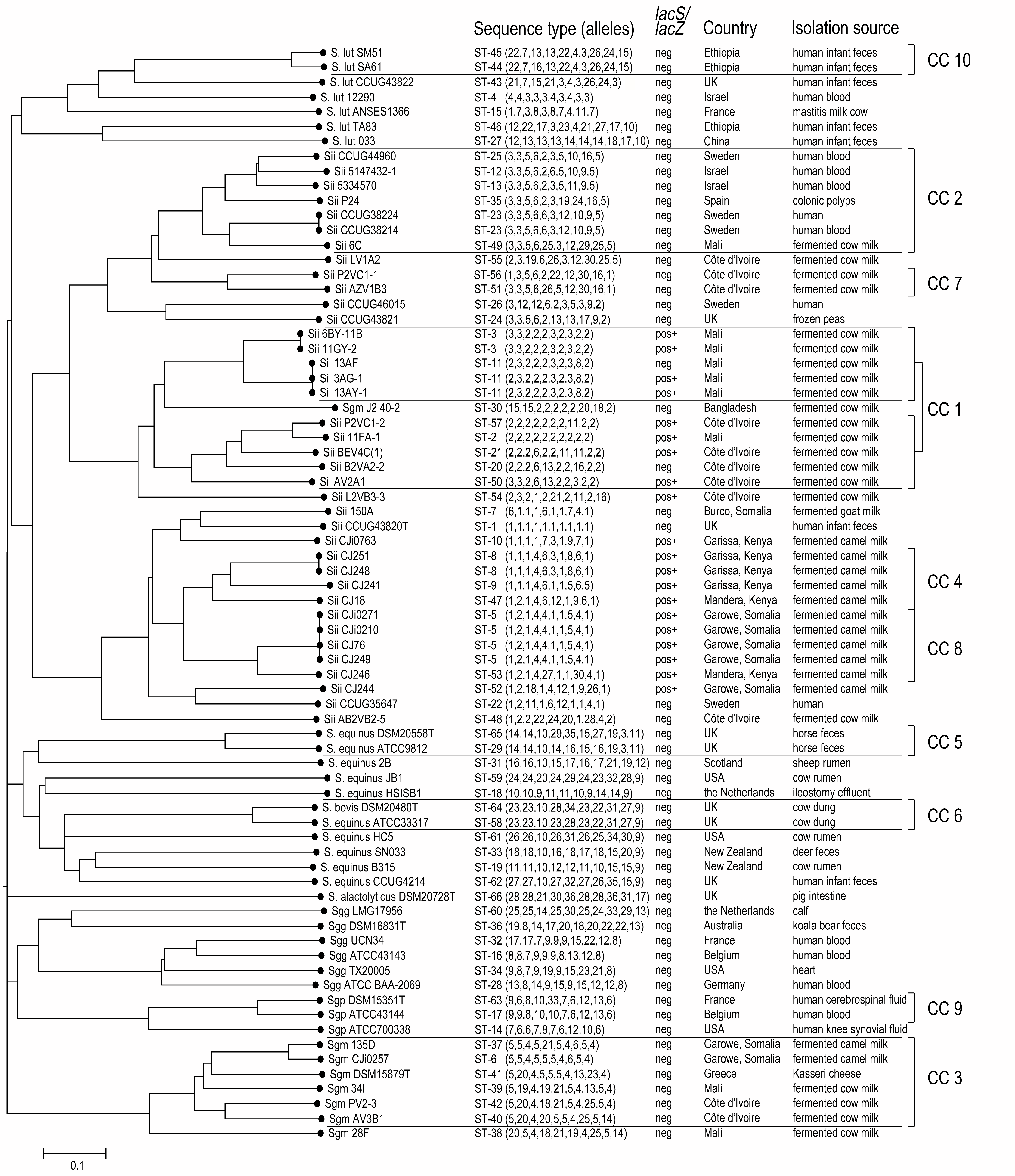

Supplement: Additional file 8: — Title of data: Profile-based MLST tree of all species within the SBSEC. Description of data: The strain pool comprises commensal animal and human strains, human pathogenic strains and food-derived strains. The tree was calculated by neighbor joining algorithm using the allele profiles of 10 housekeeping genes. Corresponding sequence types (ST), alleles and prevalence of dairy adaptation marker genes (lacS/lacZ) including origin and isolation source are indicated. Species abbreviations: S. infantarius subsp. infantarius (Sii), S. gallolyticus subsp. gallolyticus (Sgg), S, gallolyticus subsp. macedonicus (Sgm), S. gallolyticus subsp. pasteurianus (Sgp). (TIF 2600 kb) [file 12866_2016_735_MOESM8_ESM.tif]

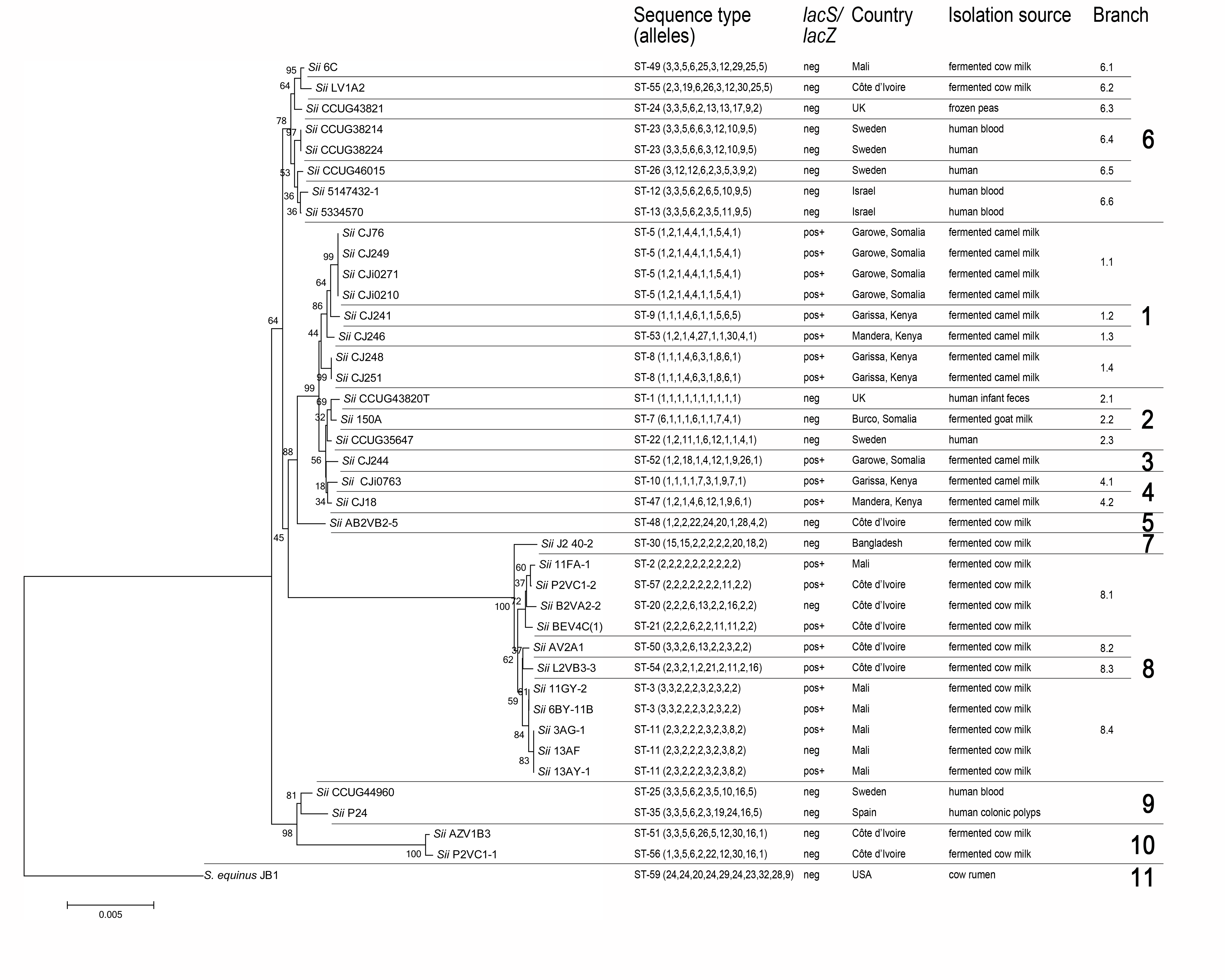

Supplement: Additional file 9: — Title of data: Sequence-based phylogenetic MLST tree of S. infantarius subsp. infantarius (Sii). Description of data: The MLST tree comprised commensal animal and human strains, human pathogenic strains and food-derived strains of all SBSEC species. The tree was calculated by neighbor joining algorithm using the concatenated partial sequences of 10 housekeeping genes. Trees were rooted to S. equinus JB1. Corresponding sequence types (ST), alleles and prevalence of dairy adaptation marker genes (lacS/lacZ) including origin and isolation source are indicated. Branches were defined and numbered according to the clonal complex specifications. (TIF 1167 kb) [file 12866_2016_735_MOESM9_ESM.tif]

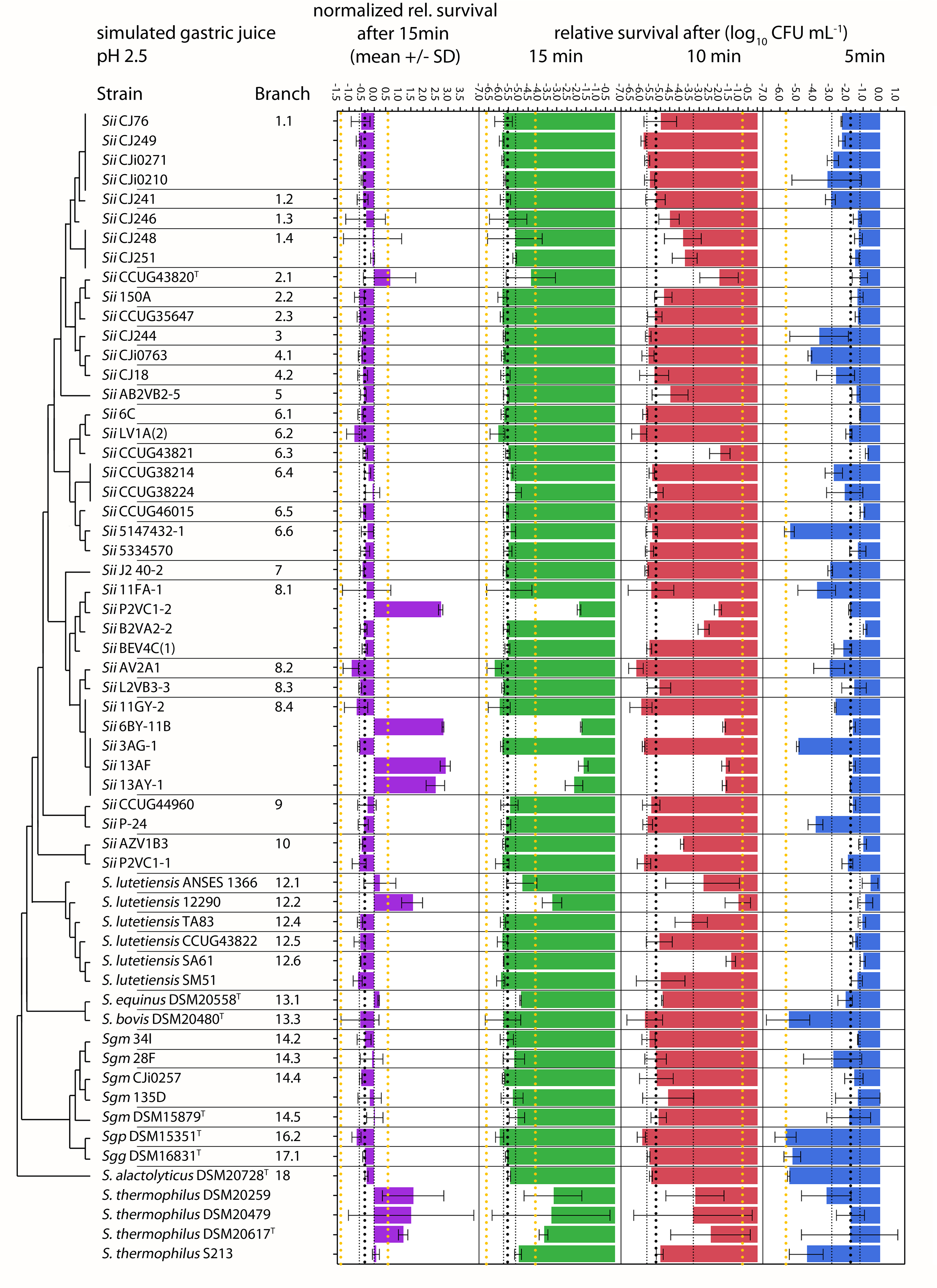

Supplement: Additional file 10: — Title of data: Relative survival of SBSEC members under simulated gastric conditions at pH 2.5. Description of data: Relative survival of SBSEC strains after 5, 10 and 15 min incubation in SGJ at pH 2.5 performed in two biological replications. Relative survival values in log10 CFU mL−1 were normalized according to the mean of all measurements of one condition to allow comparison between strains. Positive normalized values indicate relative survival higher than the mean of all strains. Statistical distribution indicators: Median: large black dotted line; Q1 and Q3: small black dotted line; lower and upper outlier fence: large yellow dotted line (only drawn if within the graph range displayed). The phylogenetic tree is based on the MLST-tree but not drawn to scale. (TIF 3894 kb) [file 12866_2016_735_MOESM10_ESM.tif]

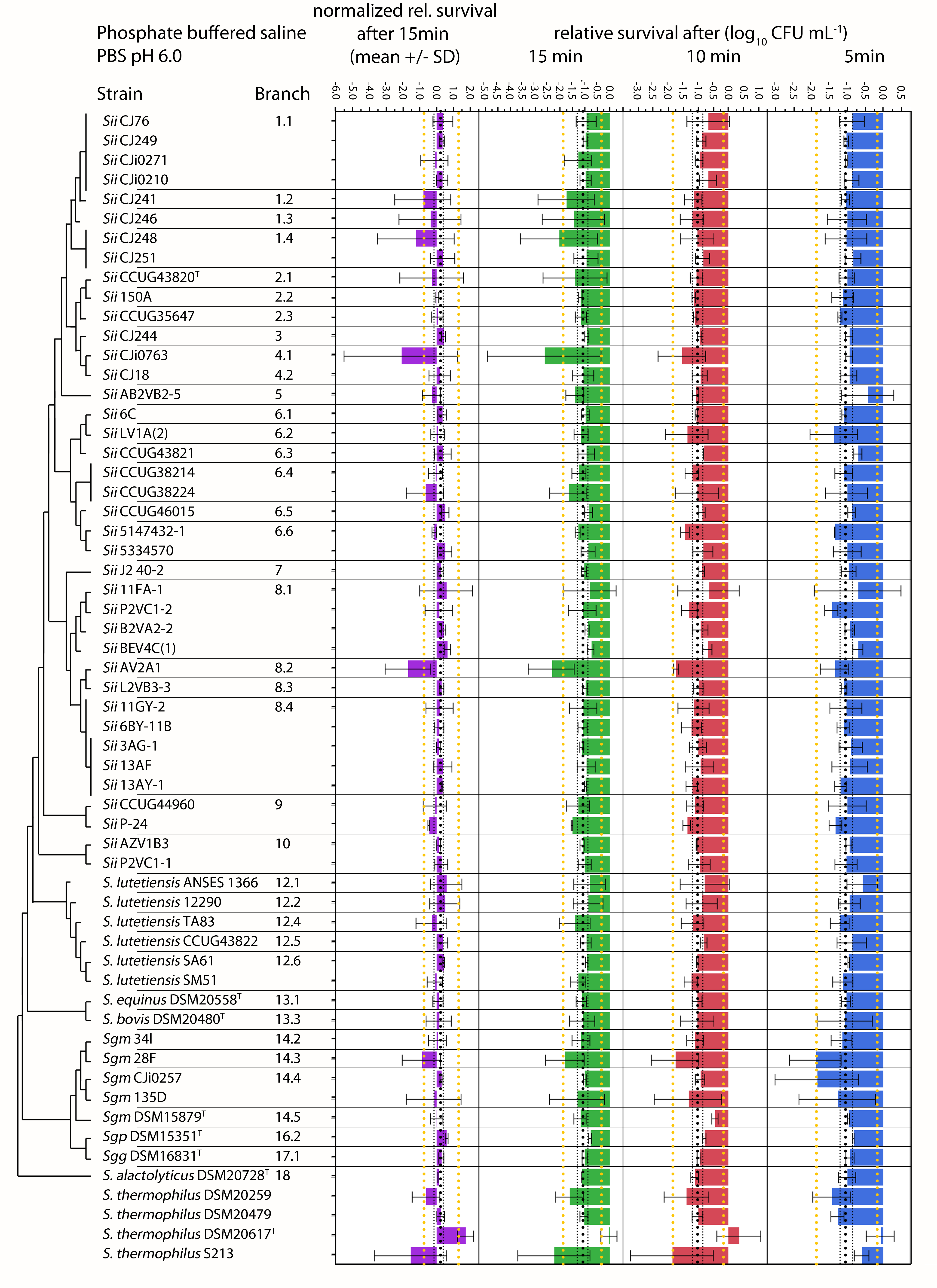

Supplement: Additional file 11: — Title of data: Relative survival of SBSEC members under simulated gastric conditions in PBS pH 6.0. Description of data: Relative survival of SBSEC strains at 5, 10 and 15 min in PBS pH 6.0 performed in 2 biological replications. Input of 0 log10 CFU mL−1 was used as basis from which relative survival is expressed. Relative survival values were normalized to allow comparison between strains where positive values indicate relative survival higher than the mean of all strains. Statistical distribution indicators: Median: large black dotted line; Q1 and Q3: small black dotted line; lower and upper outlier fence: large yellow dotted line (only drawn if within the graph range displayed). The phylogenetic tree is based on the MLST-tree but not drawn to scale. (TIF 3405 kb) [file 12866_2016_735_MOESM11_ESM.tif]

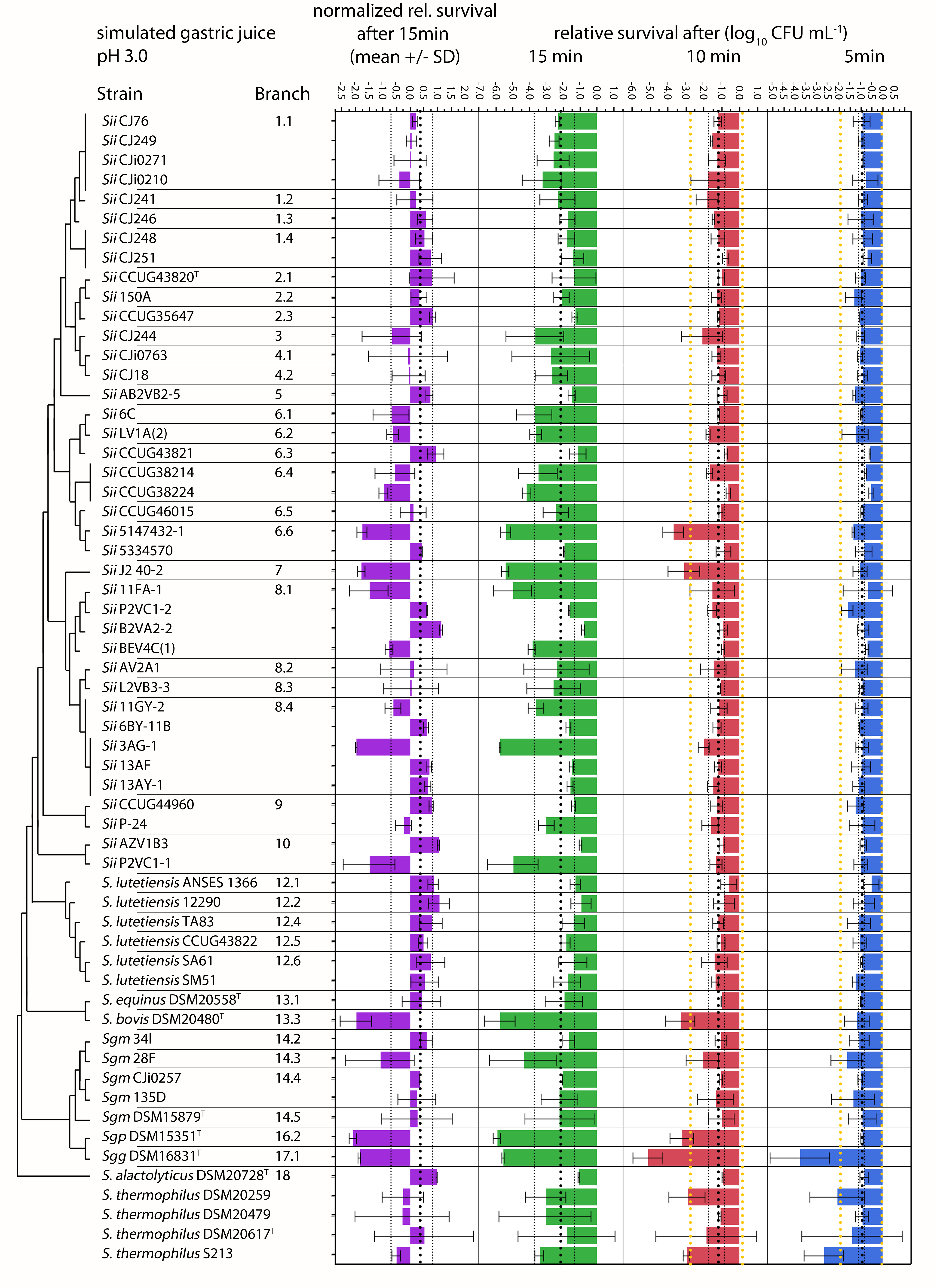

Supplement: Additional file 12: — Title of data: Relative survival of SBSEC members under simulated gastric conditions at pH 3.0. Description of data: Relative survival of SBSEC strains at 5, 10 and 15 min in in simulated gastric conditions at pH 3.0 performed in 2 biological replications. Input of 0 log10 CFU mL−1 was used as basis from which relative survival is expressed. Relative survival values were normalized to allow comparison between strains where positive values indicate relative survival higher than the mean of all strains. Statistical distribution indicators: Median: large black dotted line; Q1 and Q3: small black dotted line; lower and upper outlier fence: large yellow dotted line (only drawn if within the graph range displayed). The phylogenetic tree is based on the MLST-tree but not drawn to scale. (TIF 3007 kb) [file 12866_2016_735_MOESM12_ESM.tif]
